# Hydrocarbon Formation from Syngas with In-Operando Monitoring of Cobalt- and Manganese-Based (pre)Catalysts Using X-ray Diffraction

**DOI:** 10.1021/acsomega.4c04553

**Published:** 2024-06-25

**Authors:** Ravneet K. Bhullar, Wenqian Xu, Michael J. Zdilla

**Affiliations:** † Department of Chemistry, Temple University, 1901 N. 13th St., Philadelphia, Pennsylvania 19086, United States; ‡ X-ray Science Division, Argonne National Laboratory, 9700 South Cass Avenue, Lemont, Illinois 60439, United States

## Abstract

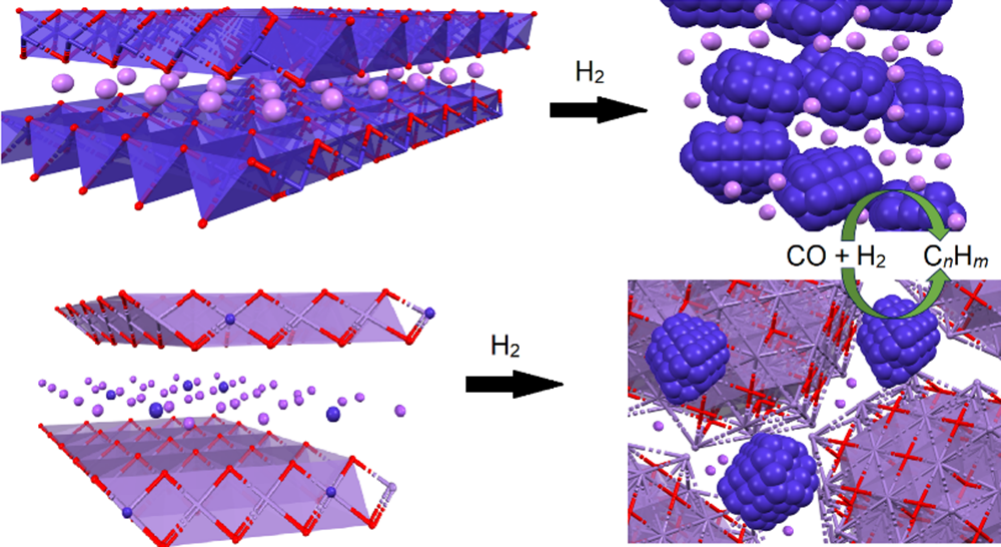

Two-layered metal oxides (LiCoO_2_ and cobalt-doped K_*n*_MnO_2_, *n* < 1) were explored as precatalysts for nanoconfined cobalt-based Fischer–Tropsch catalysts for conversion of syngas (CO and H_2_) to hydrocarbons. Ex situ, in situ, and PDF XRD analyses are presented. Based on in situ XRD analysis, LiCoO_2_ underwent reduction to predominantly cubic and hexagonal phases of cobalt metal. Reaction with syngas resulted in the generation of carbon, cobalt carbide, and lithium carbonate, in addition to the metallic cobalt phases. In the case of cobalt-doped birnessite, catalyst activation converted the birnessite phase to manganite and the cobalt to elemental cobalt, along with similar lithium and carbon phases. Conversion of syngas to C_1_ through C_7_ products was observed. The best conversions were observed for the LiCoO_2_ precursor catalyst, with generally a low olefin-to-paraffin ratio. While the conversions for the cobalt-doped birnessite precatalyst were generally lower, with lower chain lengths (up to C_5_), these catalysts gave a strikingly high olefin-to-paraffin ratio: in the best case, greater than 20:1.

## Introduction

The world’s reliance on conventional fossil fuels as the primary source of energy puts our planet in peril. The dwindling oil reserves, high cost of purification to remove sulfur and other harmful impurities, and environmental concerns from the continued emission of CO_2_ have stimulated energy diversification and encouraged researchers to explore and develop alternative routes to produce hydrocarbons of diverse chain lengths from non-oil feedstocks.^1^ Indirect gas-to-liquid (GtL), coal-to-liquid (CtL), and biomass-to-liquid (BtL)—collectively referred to as XtL—processes enable feedstock expansion and production of “ultraclean” fuels. The second and the most important stage of XtL processes is the Fischer–Tropsch synthesis (FTS), involving the catalytic conversion of syngas (CO and H_2_) into a range of hydrocarbon products. The technology and techniques required for refining these product mixtures depend on the nature of products synthesized during the FTS step.^2^ The main products of the FTS reaction include light olefins, higher hydrocarbons, methanol, ethylene glycol, dimethyl ether, and aromatics. Light olefins (C_2_–C_4_) are the key building blocks of the chemical, plastic, coating, and cosmetic industries. The synthetic liquid fuels synthesized via the FTS process have a very low sulfur and aromatic content compared to gasoline or diesel from crude oil.^3^

A variety of catalytic systems have been developed by researchers that exhibit a CO hydrogenation activity. Metals such as iron, cobalt, nickel, and ruthenium have been found to be sufficiently active for these applications. Iron and cobalt are more favorable catalysts from a commercial standpoint due to their low cost and high availability. Nickel forms volatile nickel carbonyl at the operating conditions of the FTS plant and produces much more methane than Fe or Co.^4^ The activity of FTS catalysts can be further enhanced by adding chemical promoters and can improve mechanical stability. The surface area of the active metal can be extended by dispersing it on a support or carrier material.^5^

**Figure 1:**
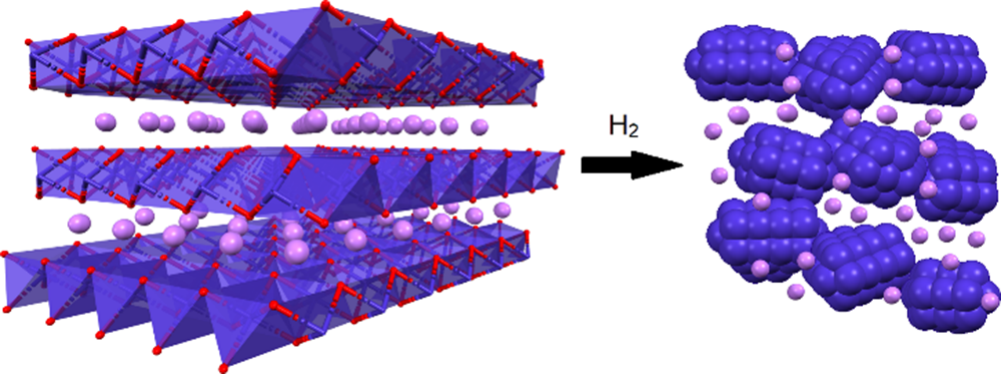
Conceptual illustration of the formation of a lithium–cobalt-based FTS catalyst formed from reduction of layered LiCoO_2_. Blue spheres represent cobalt atoms, and purple spheres represent Li^+^ ions.

In this study, we aimed to explore the catalytic activity of a cobalt-based catalyst synthesized from layered LiCoO_2_ supported on silica as a precatalyst for the Fischer–Tropsch synthesis. The Co (II) and Co (III) ions in LiCoO_2_ were reduced to metallic cobalt during the activation phase. Due to the layered morphology of LiCoO_2_ (Figure 1), we predicted that the reduction of the layered LiCoO_2_ material to metallic cobalt mixtures would lead to the development of novel small-particle morphology and nanoconfinement of substrate and active catalytic species, which can enhance catalysis.^6^ The presence of alkali cations in the precatalyst is expected to provide cationic lithium sites that could promote the CO dissociation through polarization of the CO bond via cooperative Lewis acid catalysis.^7^

Previous studies have suggested that MnO serves as a support for metallic Co nanoparticles, thereby ensuring a good dispersion and stabilization of these nanoparticles.^8^ MnO is also known to act as an electronic and structural promoter, and the promoting effects of MnO are strongly dependent on its location and amount. Morales et al. showed that CO preferentially bonded linearly to surface metal sites when the MnO loading was much lower than the Co loading. We therefore sought to generate and monitor in situ a similar catalytic system that would suspend the cobalt nanocatalyst within a crystalline Mn–O lattice.

In this report, we evaluate the catalytic activity of the reduced product of the layered LiCoO_2_ precatalyst for FTS and monitor the phase speciation of the catalyst in situ using XRD. The layered precatalyst is activated by reduction with hydrogen gas at typical FTS temperatures of 200 °C as well as elevated temperatures, in order to explore the effects of temperature on catalysis at temperatures between 200 and 500 °C. Furthermore, to examine the role of catalyst confinement in a noncatalytic medium, we also explored the formation of this catalyst from a layered, cobalt-doped manganese oxide precursor, which gave cobalt nanocatalysts dispersed in a less reactive Mn–O crystal matrix. Product selectivities are reported and contrasted for individual temperatures, and catalyst speciation during the reaction is described.

## Experimental Section

### General Considerations

All chemicals were purchased from commercial sources and used without further purification. Transmission electron microscopy (TEM) images were collected using a JEOL JEM-1400 microscope operating at 120 kV. Scanning electron microscopy and energy dispersive spectroscopy (SEM-EDS) samples were deposited on conductive carbon tape mounted on aluminum stubs, and images were collected on an Agilent 8500 FE-SEM instrument operating at 1 kV. The elemental content was analyzed using SEM-EDS. X-ray photoelectron spectroscopy (XPS) was performed using a Thermo Fischer K-alpha+ instrument with an Al k-α monochromatic X-ray source at The University of Delaware. The collected data were processed by using Casa XPS software. To determine the volume-specific surface area (VSSA), measurements of surface areas were carried out by nitrogen adsorption with the application of Brunauer–Emmett–Teller (BET) analysis using a Quantachrome Quadrasorb SI analyzer. Elemental analysis was performed using a Thermo Scientific iCAP 7000 Series ICP-OES.

### Synthesis

The lithium cobalt oxide, LiCoO_2_, was synthesized by following the procedure described in a previous study.^9^ Cotton was soaked in an aqueous solution of 0.5 M lithium nitrate (LiNO_3_) and 0.5 M cobalt nitrate Co(NO_3_)_2_ for 3 h. After soaking, the cotton was squeezed to remove excess solution, heated in a furnace to a temperature of 400 °C at the rate of 100 °C/h, held at this temperature for 8 h, and then allowed to cool to room temperature. The resultant material was ground with a mortar and pestle and reheated in the furnace to 900 °C at the rate of 100 °C per hour. The final product was cooled to room temperature, washed with deionized water, and collected via repeated centrifugation/rinse cycles until supernatant rinses were colorless and clear.

Cobalt-doped birnessite was prepared according to the published protocol.^10^ A 30% loading of birnessite with cobalt was used. ICP-OES on an acid-digested sample of cobalt-doped birnessite gave 0.327 [Co] and 0.808 [M] Mn, indicating a 28:72 Co:Mn ratio. Potassium content was derived from the EDS analysis of grains (Figure S20). The data establish that the formula as ICP-OES was K_0.08_Co_0.28_Mn_0.72_O_2_.

### FTS Catalysis

The reaction was conducted in a home-built stainless-steel U-tube reactor (Figure S1). The reactor consists of a 20 cm length of stainless-steel tubing with an inner diameter of 3.2 mm. The reactor was immersed in a sand bath to regulate the temperature of the reaction. The catalyst was loaded in the middle position of the reactor and sealed with a glass wool. The temperature of the catalyst bed was monitored by using a Cr–Al thermocouple. Syngas (H_2_/CO = 2) was metered through mass flow controller at the flow rate of 50 mL/min. The reactions were carried out at 1 atm of syngas. The reaction was performed with periodic increase in the temperatures from 200, 250, 300, 350, 400, 450, and 500 °C for 2 h each. The hot products in the effluent gas were trapped in a nitrogen cold trap. The products were extracted into dichloromethane and analyzed by an Agilent 6890N gas chromatograph with a flame ionization detector (FID) with He as the carrier gas. The products were analyzed using a nonpolar fused silica PLOT column (Restek Rt-Q- Bond column, see Table S1 for column details and methods). Repeated experiments carried out under the same reaction conditions demonstrated that the studied catalyst showed good reproducibility.

For a typical FTS reaction, the CO conversion and product selectivity were calculated by the equations:
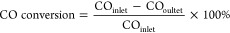
1
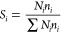
2where CO_inlet_ and CO_outlet_ represent moles of CO at the inlet and the outlet, respectively. *S*_*i*_ denotes the selectivity to product *i* on an atomic carbon basis, *N*_*i*_ is the molar fraction of product *i*, and *n*_*i*_ is the carbon number of product *i*.

### Powder X-ray Diffraction Analysis

The ex situ powder X-ray diffraction (PXRD) patterns were performed on a Bruker Kappa APEX II DUO diffractometer and Mo kα radiation from a sealed tube and processed using the DIFFRACEVA software package. The patterns were indexed to the Crystallography Open Database (COD).

In-situ PXRD experiments were carried out at the 17-BM beamline at Advanced Photon Source (APS) at Argonne National Laboratory (ANL) using X-ray wavelength 0.24105 Å and a miniature reactor.^11^ Sample powder was packed in a 1.1 mm diameter fused silica capillary connected to the reactor cell. The sample was first reduced with 5% hydrogen (helium balanced) at 200 °C for 30 min. FTS reaction was mimicked with 5% syngas (H_2_:CO = 2:1, He balanced). The sample was heated from room temperature to 500 °C at a heating rate of 10 °C/min under a flow rate of 50 mL/min. The reaction was stabilized at the temperatures of 200, 250, 300, 350, 400, 450, and 500 °C for 2 h for the collection of XRD patterns. Two-dimensional (2D) diffraction data were collected with a VAREX flat panel amorphous Si detector in X-ray transmission geometry. Reduction of the 2D images to a 1D XRD pattern of intensity versus 2Θ was carried out using the GSAS-II program.^12^ GSAS-II was also used for subsequent Rietveld analysis. Phase identification was done with the Inorganic Crystal Structure Database (ICSD) and the Mercury software.^13^ The instrumental parameters used for the Rietveld refinement were determined from a NIST LaB_6_ calibrant.

## Results and Discussion

### Precatalyst Characterization

**Figure 2:**
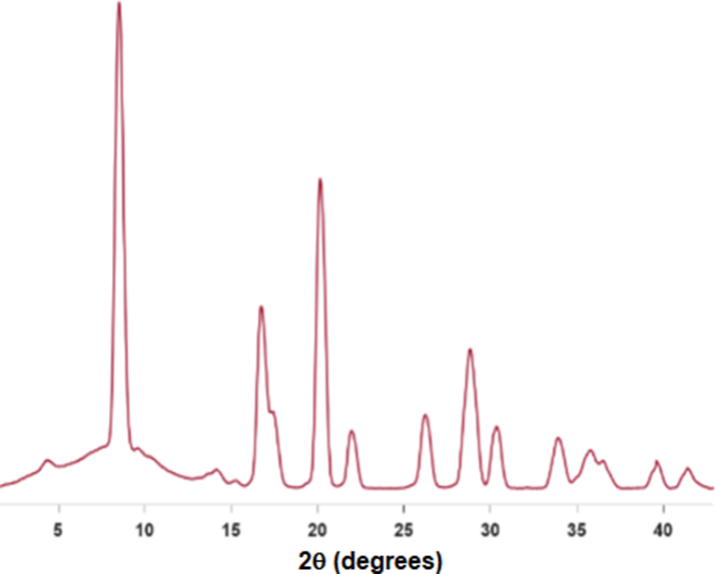
PXRD of LiCoO_2_ using MoKα radiation.

The PXRD pattern (Figure 2) of LiCoO_2_ was consistent with the previously reported data.^9^ Hexagonal unit cell dimensions are *a* = 2.8156 Å, *b* = 2.8156 Å, *c* = 14.0542 Å, α = 90°, β = 120°, and γ = 90°. LiCoO_2_ crystallizes in a rhombohedral *R3m* structure. The unit cell in the hexagonal setting contains three formula units. The stacked layers are composed of cobalt octahedrally surrounded by edge-sharing oxygen and intercalated by Li^+^ ions (Figure 1).^14^

**Figure 3:**
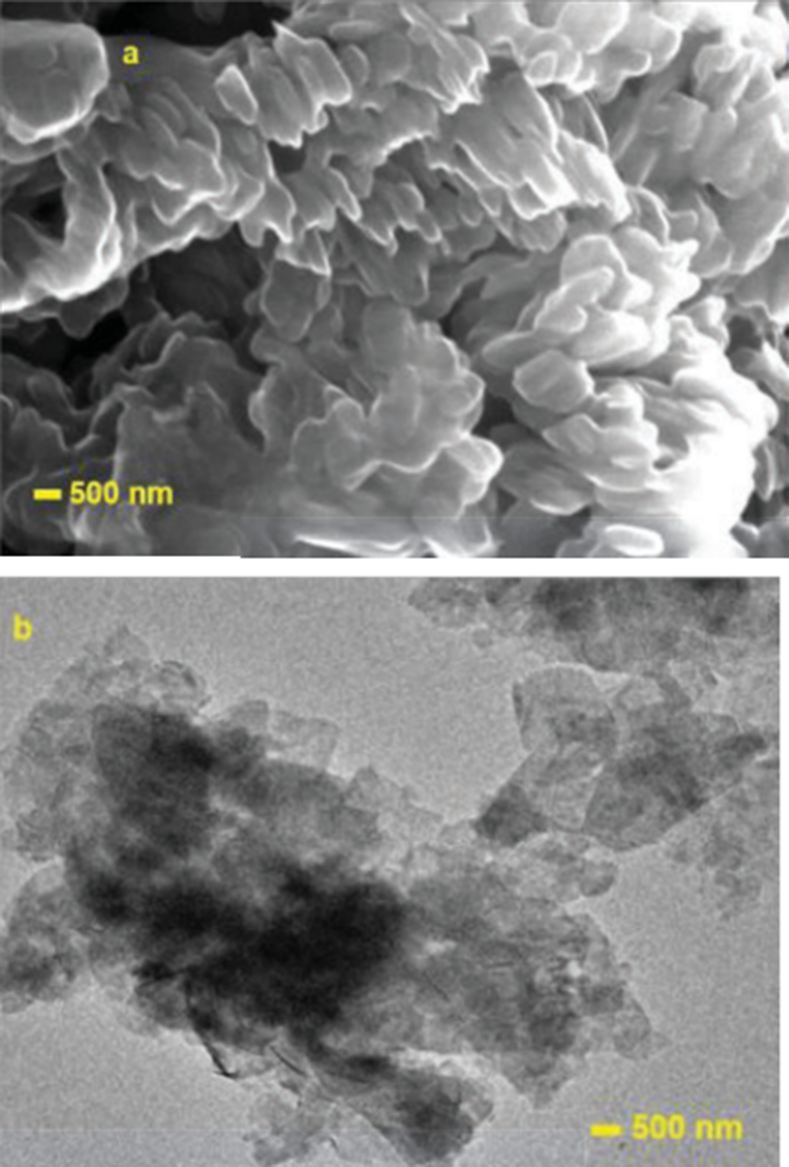
(a) SEM and (b) TEM images of LiCoO_2_.

The SEM and TEM images (Figure 3) confirmed the layered morphology of the samples. The elemental composition was confirmed by SEM-EDS spectra, as shown in Figure S2. The X-ray photoelectron spectrum (XPS) plot in Figure S3 showed a Co-2p_3/2_ peak at 780 eV and a Co-2p_1/2_ peak at 795 eV. The strong broadening of the peaks can be attributed to the presence of Co^2+^ ions in the oxygen environment in addition to Co^3+^.^14^ BET (Figure S6) indicates a volume-specific surface area of 72.75 m^2^/cm^3^.^15^

**Figure 4:**
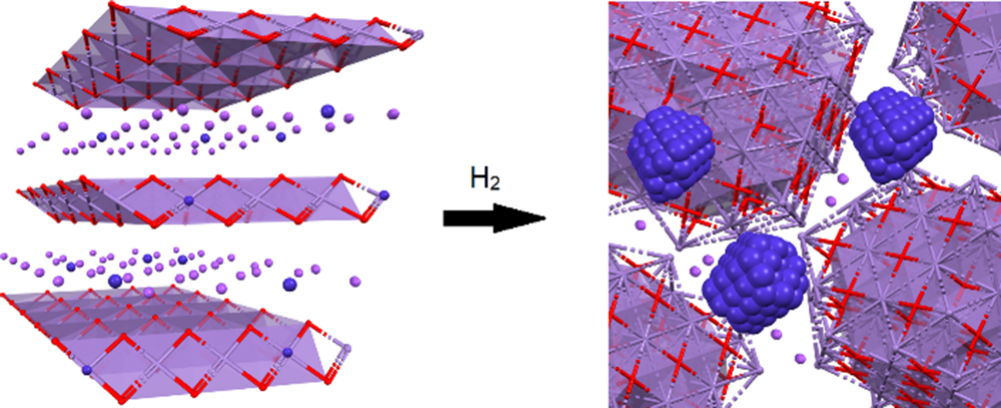
Conceptual illustration of the formation of nanostructure potassium–cobalt-based FTS catalyst within a Mn–O matrix, formed from reduction of cobalt-doped layered KMnO_2_. Blue ions represent cobalt, purple polyhedra represent the Mn–O lattice, and purple spheres represent K^+^ ions.

The preorganization of nanoconfined environments was a goal of the use of layered precatalysts with interlayer ions. However, the cobalt ions exclusively became elemental cobalt during the activation phase. In order to explore the effect of continued nanoconfinement of the cobalt catalyst, we performed a comparative study, where a cobalt-doped birnessite (layered potassium–manganese oxide)^10^ was used as the precatalyst. This material possesses cobalt ion substitutions in both the layered metal oxide lattice, as well as the interlayer (Figure 4).^10^ In this material, the expectation is that the activation step with H_2_ will reduce the cobalt ions to elemental cobalt and the manganese oxide system to a Mn(II)-based lattice. This should provide particulate cobalt catalysts isolated in confined spaces between Mn^II^O crystals.

**Figure 5:**
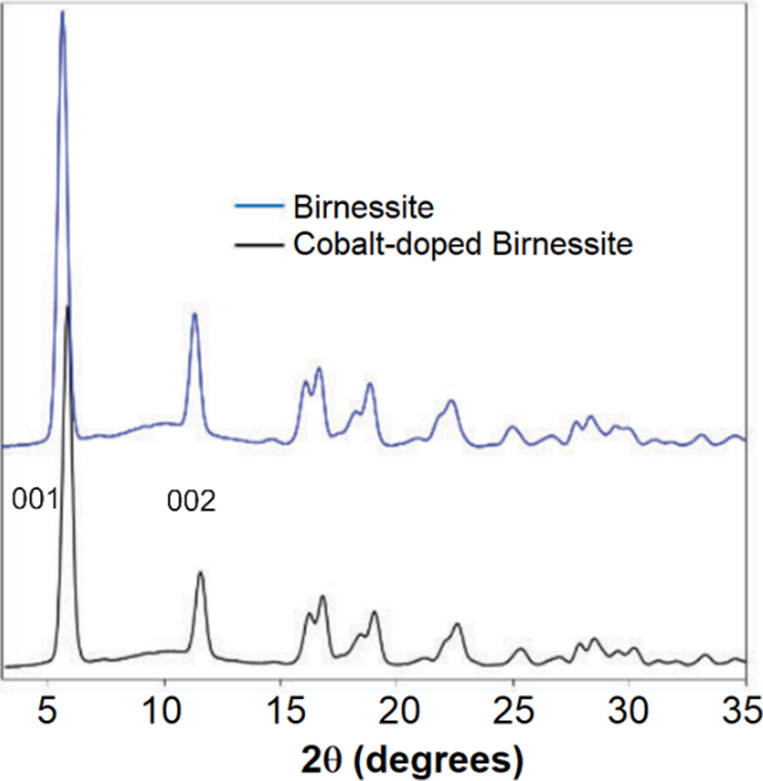
PXRD of cobalt-doped birnessite using MoKα radiation.

The PXRD pattern of Co-doped birnessite is shown in Figure 5. The pattern was consistent with the previously reported data of birnessite. The decrease in the intensity of (001) and (002) peaks and concurrent peak broadening (in comparison to parent birnessite) indicated a decrease in crystallinity because of cobalt doping. Additionally, the peaks shifted to a higher angle, suggesting a decrease in the crystal size in c-direction as the d-value decreased from 7.33 to 7.23 Å.^11^ This suggests an overall increase in the charge of the sheets due to interlayer Co^2+^ ions, which, in turn, enhances the ionic attraction between the sheets and intercalated divalent cobalt cations, thereby reducing the interlayer distance.

**Figure 6:**
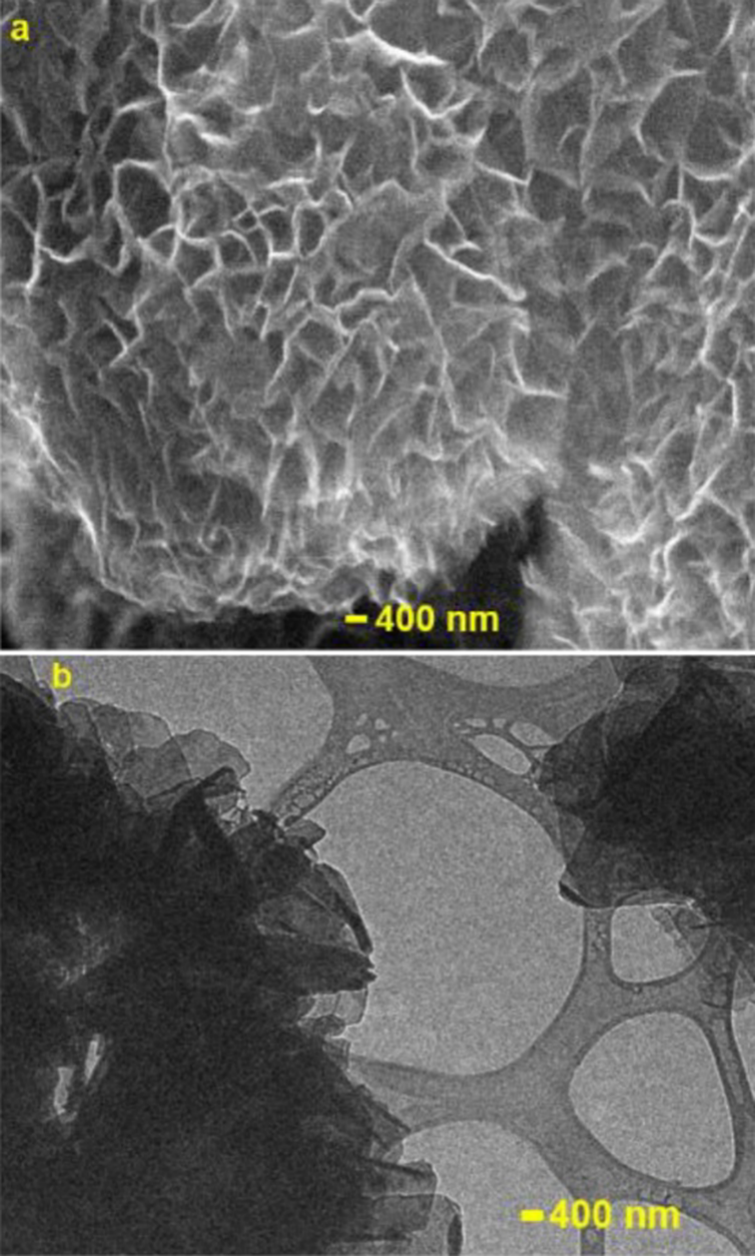
(a) SEM (b) and TEM images of Co-doped Birnessite.

The SEM and TEM images (Figure 6) indicated that the layered flower pod morphology was preserved after cobalt doping into birnessite. The elemental composition was confirmed by the SEM-EDS spectrum, as shown in Figure S4. A high-resolution X-ray photoelectron spectrum was obtained and fitted for the determination of average oxidation state using the approach of Nesbitt.^16^ The Mn atoms were present in mixed oxidation states II, III, and IV with an average oxidation state of 3.57. The X-ray photoelectron spectrum (XPS) plot showed a Co-2p^3^/_2_ peak at 780 eV and a Co 2p1/2 peak at 795 eV (Figure S5).

The BET adsorption isotherm in Figure S7 was identified as a type I isotherm, highlighting microporosity and therefore high surface area. The type I isotherm is characterized by a high uptake of nitrogen at low relative pressures (*P*/*P*_0_ < 0.2) resulting from the filling of micropores. After filling the pores, the material does not absorb a significant amount of gas until capillary condensation occurs at around *P*/*P*_0_ = 1. The BET surface area of Co-doped birnessite was 117.6 m^2^/g. The measured volume-specific surface area was 39.2 m^2^ cm^–3^.

### Catalytic Performance

The products were determined by analyzing the outlet gases by offline GC-FID and GC-TCD. Table 1 summarizes the catalytic performance and product distribution for FTS reaction over LiCoO_2_ in the temperature range of 200–500 °C. The catalyst was exposed to a syngas mixture (H_2_:CO = 2:1) at the flow rate of 3000 mL/h/g. Overall, the catalyst exhibited a high %CO conversion. The CO_2_ selectivity was low up to 350 °C (3.9%C). With a further increase in temperature, CO_2_ selectivity (42.6%C) increased due to enhanced water–gas shift activity.^32^ Generally, the methane selectivity decreased with increasing temperature. The sudden change in activity at 400 °C denotes a change in equilibrium speciation at the catalyst and may be attributable to sintering of catalyst particles (see Discussion section).

**Table 1:** Catalytic Performance and Product Distribution for FTS Reaction Using LiCoO_2_ Precatalyst in the Temperature Range 200–500 °C

temperature (°C)	conversion (%)	selectivity (%) CO_2_	CH_4_	C_2_	C_3_	C_4_	C_5_	C_6_	C_7_
200	65.7	1.5	23.31	33.23	8.22	8.71	4.02	7.34	4.39
250	66.2	0.98	18.54	32.6	13.4	13.94	7.09	7.74	2.21
300	92.9	2.09	2.43	19.88	20.2	43.8	9.54	2.15	0.62
350	80.4	3.95	12.64	20.36	7.21	15.8	2.25	0.69	1.49
400	26.7	42.6	6.48	31.6	8.74	7.72	3.3	0.69	0.56
450	78.5	45.2	7.46	20.7	8.19	8.29	4.08	2.65	3.36
500	83.7	58.7	12.45	20.87	1.48	2.3	0.49	2.89	0.41

**Figure 7:**
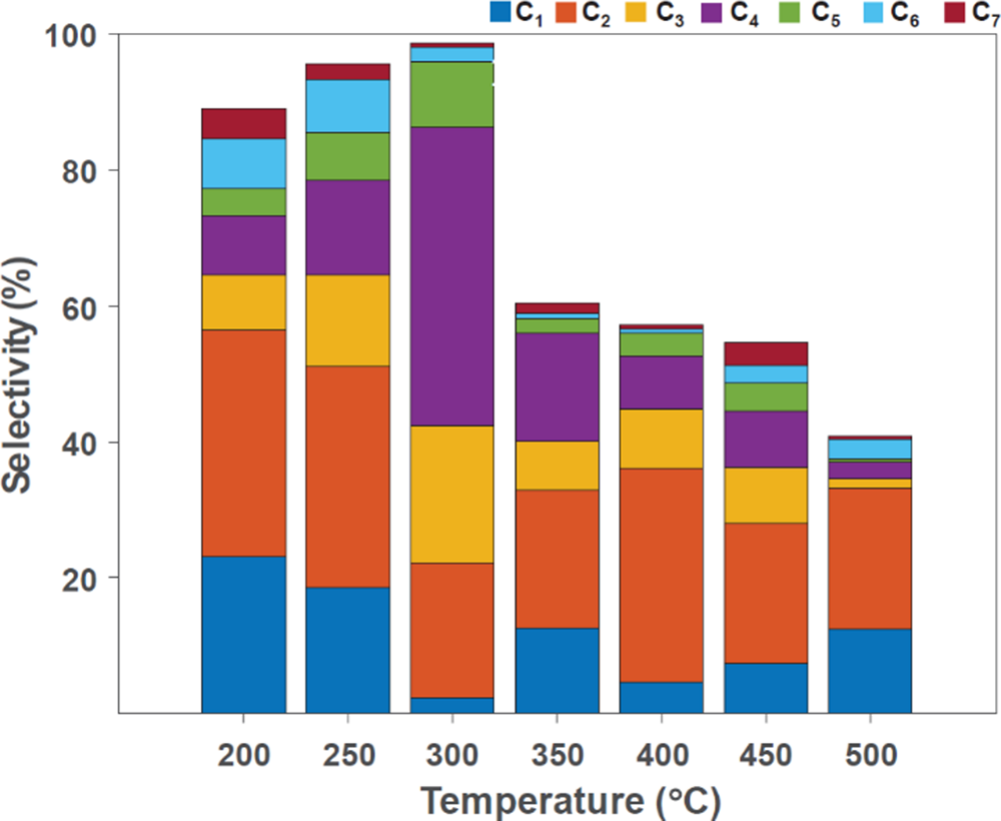
Product distribution as a function of temperature using a LiCoO_2_ precatalyst.

**Figure 8:**
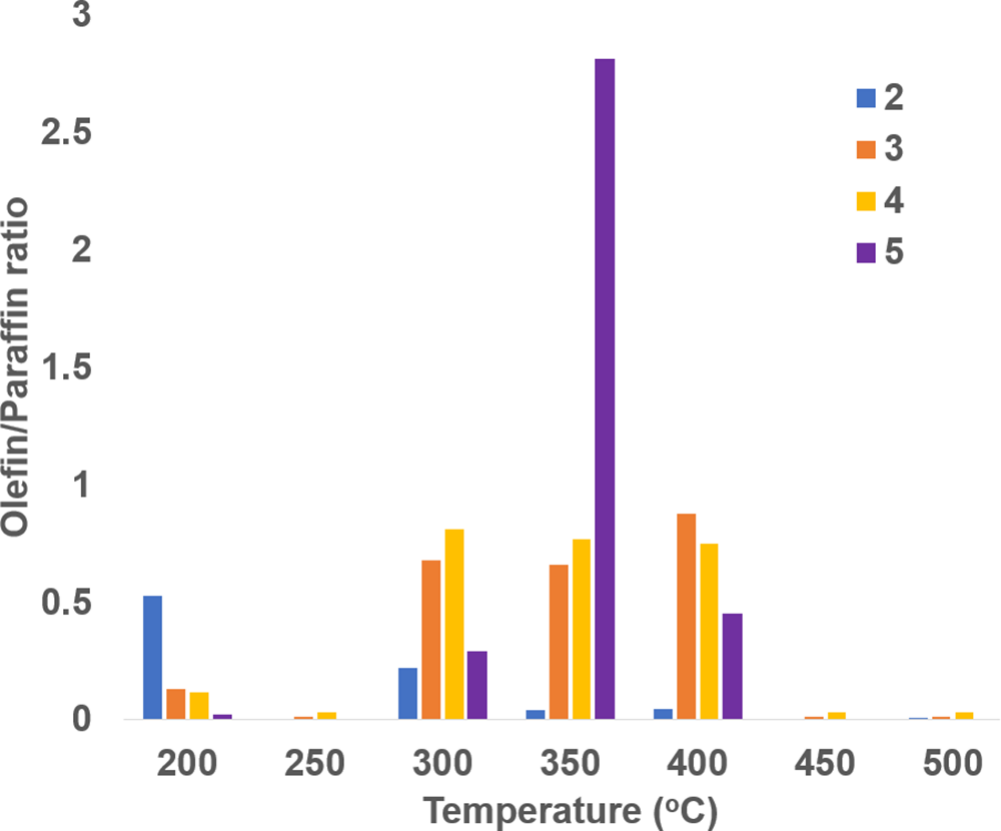
Olefin/paraffin ratio as a function of temperature resulting from FT using the LiCoO_2_ precatalyst.

A comparison of the product distribution at all operating temperatures is described in Figure 7 and Table 1. The C_2_ products exhibited the highest average selectivity of 25.6%C, followed by C_4_ with an average selectivity of 14.40%C. At 300 °C, C_4_ products dominated the distribution with 43.8%C. The overall product yield decreased with a further increase in temperature due to the increase in the water gas shift activity and a corresponding increase in the production of CO_2_. Figure 8 shows the olefin/paraffin ratios in the reaction temperature range. The catalytic results indicated that 1,3-butadiene (12.05%C) was the most abundant product in the olefin distribution, followed by propylene (9.3%C). Paraffins constituted the main products at all of the temperatures. The o/p ratio generally decreased with an increase in temperature (Table 2 and Figure 8).

**Table 2:** Olefin/Paraffin Ratio as a Function of Temperature

carbon count (*n*_*i*_)	olefin to paraffin (o/p) ratios						
	200 °C	250 °C	300 °C	350 °C	400 °C	450 °C	500 °C
2	0.53	0.0049	0.22	0.044	0.046		0.007
3	0.13	0.015	0.68	0.66	0.88	0.014	0.014
4	0.12	0.034	0.81	0.77	0.75	0.033	0.031
5	0.023		0.29	2.81	0.45		

Information about the mechanism of polymerization FTS may be gleaned from examination of the chain growth probability as a function of temperature. Anderson–Shultz–Flory (ASF) plots of ln(mol) fraction of product vs number of carbons give a slope (α), approximating the chain growth probability resulting from a hydrocarbon growing by combination with a nearby one-carbon unit at the catalyst surface. In this work, generally, the chain growth probability (α) values decreased with an increase in temperature (Figure S8). However, ASF plots shown in part S8 show significant scatter, indicating deviations from ideal ASF behavior. This is typical of heterogeneous catalytic reactions as the kinetic parameters such as temperature, H_2_/CO ratio, partial pressures of the individual reagents, and reaction products cannot be kept identical and constant in the immediate proximities of all catalytic sites throughout the reactor via a dynamic steady state equilibrium. In other words, the rate of reagent chemisorption of the gas molecules is much faster than the rates of supply by chain transport.^17^

The mixed manganese–cobalt system shows a similar product range as the all-cobalt system but with some differences. In general, this dispersed catalyst system gave shorter-chain alkanes but a much higher olefin:paraffin ratio. The product analysis suggested that the reaction exhibited low %CO conversion (average of 26.4% CO conversion) up to 300 °C. At 350 °C, the %CO conversion increased to an average of 95%. The overall %CO conversion was comparable to that of previously explored catalysts such as MnO_*x*_/Co, Co_1_Mn_3_–Na_2_S and, Co3Mn_1_–Na_2_O.^18^

**Figure 9:**
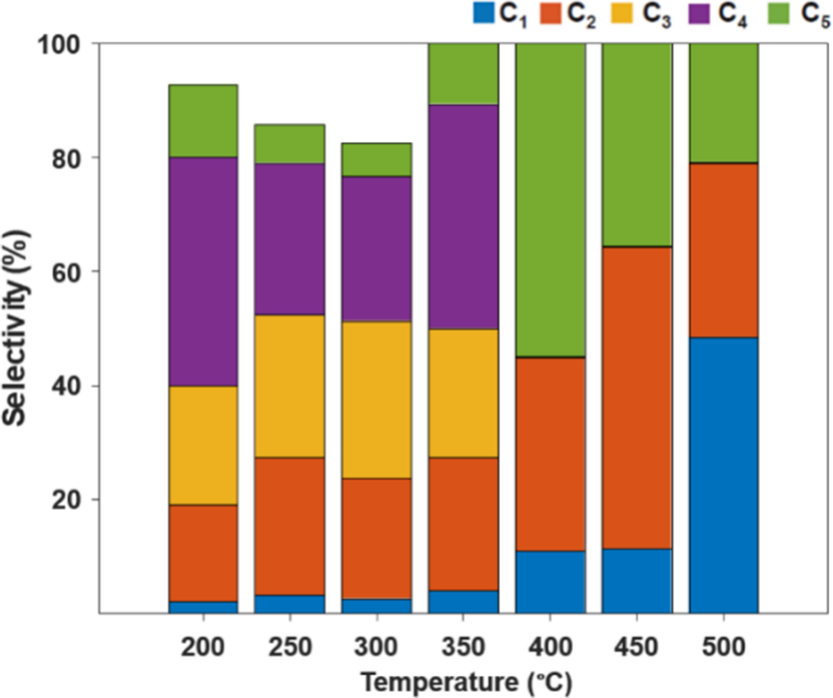
Product distribution as a function of temperature using the Mn–Co bifunctional catalyst.

A comparison of product distribution at all operating temperatures is described in Figure 9 and Table 3 and shows some distinctions from the all-cobalt system. An overall trend for the mixed Co–Mn catalyst is a decrease in the formation of longer-chain hydrocarbons. The C_4_ products exhibited the highest selectivity with an average of 32.9%C. At 400 °C, C_2_ products dominated the distribution with an average of 39.2%C. The methane selectivity increased with an increase in temperature with a simultaneous decrease in the concentration of C_2_–C_4_ products. Methane selectivity was low up to 350 °C (2.86%C), which is lower than previously studied catalysts such as MnO_*x*_/Co and BDO/MnO_*x*_/Co (1,4-butanediol, BDO).^19^ Methane selectivity increased to ∼10% at 400–450 °C and 48.35% at 500 °C. The total product selectivity at 200–300 °C was 75.9% (C_2_–C_4_), which increased to 85.50% at 350 °C. C_3_–C_4_ products were not observed above 350 °C, decreasing the product selectivity to an average of 40% (C_2_) at 400–500 °C.

**Table 3:** Catalytic Performance and Product Distribution for FTS Reaction Using Co-Doped Birnessite Precatalyst in the Temperature Range 200–500 °C

temperature (°C)	conversion (%)	selectivity (%)				
		CO_2_	CH_4_	C_2_	C_3_	C_4_
200	28.7	12.7	2	17.02	20.8	40.21
250	31	6.7	3.11	24.15	25.05	26.7
300	19.6	5.9	2.52	21.2	27.55	25.3
350	99.2	10.6	3.83	23.41	22.68	39.4
400	92.4	55	10.87	34.09		
450	91.9	35.6	11.26	53.06		
500	96.1	20.9	48.35	30.7		

Product analysis suggested that MnO/Co displayed higher selectivity toward lower olefins and low selectivity toward undesired methane and lower paraffins (Figure 9 and Table 3). This suggests that β-H elimination was the dominant termination pathway, and secondary hydrogenation of olefins was also suppressed,^19^ leading to shorter chains, but greater olefinic content. Whereas the all-cobalt system gave olefin:parrafin ratios between 0.1 and 3, the Mn–Co catalyst system gave olefin:paraffin ratios larger than 20 in some cases. CO_2_ selectivity was low (8.9%C average) up to 350 °C, which increased to an average of 37.1%C at 400–500 °C. This shift in the product selectivity and concurrent rise in %CO_2_ selectivity can be attributed to the enhanced water gas shift (WGS) reaction at higher temperatures and may be attributed to catalyst particle sintering (see Discussion section).

**Figure 10:**
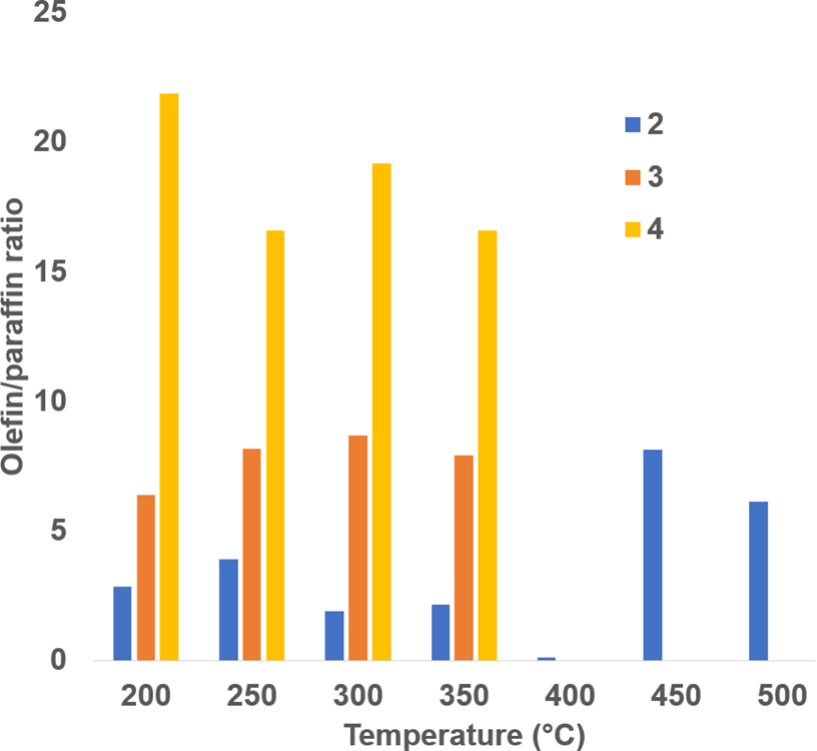
Olefin/paraffin ratio as a function of temperature from the Mn–Co bifunctional catalyst.

### In Situ Catalyst Speciation and Phase Refinement

**Figure 11:**
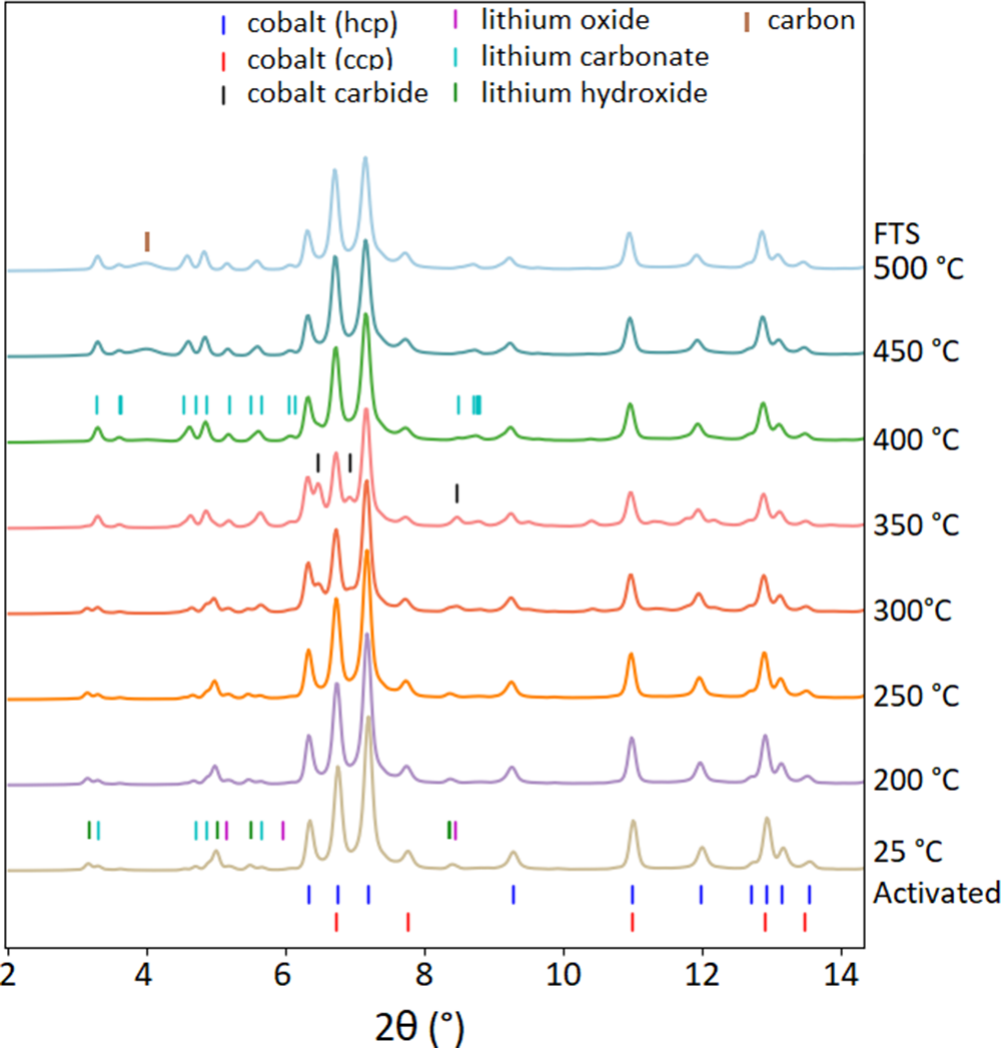
Evolution of the diffractograms of the activated LiCoO_2_ sample during the FTS catalysis process with increasing temperature.

A plot of in situ X-ray powder diffractograms obtained of the all-cobalt catalyst during the FTS catalysis at increasing temperatures can be seen in Figure 11. The sample prior to activation was mainly LiCoO_2_ (hR) with a minor component of Li_2_CO_3_ (Figure S9). The activation transformed the sample to a mixture of metallic cobalt phases (Co(hcp) and Co(fcc)) and several lithium containing phases, including Li_2_O, LiOH, and Li_2_CO_3_. The crystalline phase abundances during reduction and catalysis were analyzed with Rietveld refinement, and the results are shown in Figure S10 and Table S2. The phase speciation over time is shown using PDF analysis in Figure S18. Co(hcp) was the dominant phase of the metallic cobalt evolved during the reaction. The phase fraction of Co(hcp) was nearly 4 times that of Co(fcc) in the activated sample. Both phases had a size of ∼20 nm based on the fitting of the peak profiles. A cobalt carbide phase (Co_2_C) appeared at 300 °C at the consumption of both metallic Co phases. The carbide phase reached its maximum relative abundance of 27 wt % at 350 °C, and then started decreasing and was no longer observed at 450 °C, whereas the metal Co phase fractions rebounded. The Co(fcc) phase was slightly favored over the Co(hcp) phase in the carbide to metal transition. Carbide is implicated in some FTS mechanisms,^20^ but also can serve to inhibit others.^21^ The concentration of the Co(fcc) phase at 500 °C was 26 wt % of all the cobalt-bearing phases, greater than the 22 wt % in the activated sample (Figure S10). Also starting at 400 °C, carbon accumulation was observed, indicated by the growing hump centered at a 2θ angle of 4° from 400 to 500 °C. The carbon phase has an estimated crystallite size of 4 nm based on the peak profile fitting of the Rietveld analysis.

**Figure 12:**
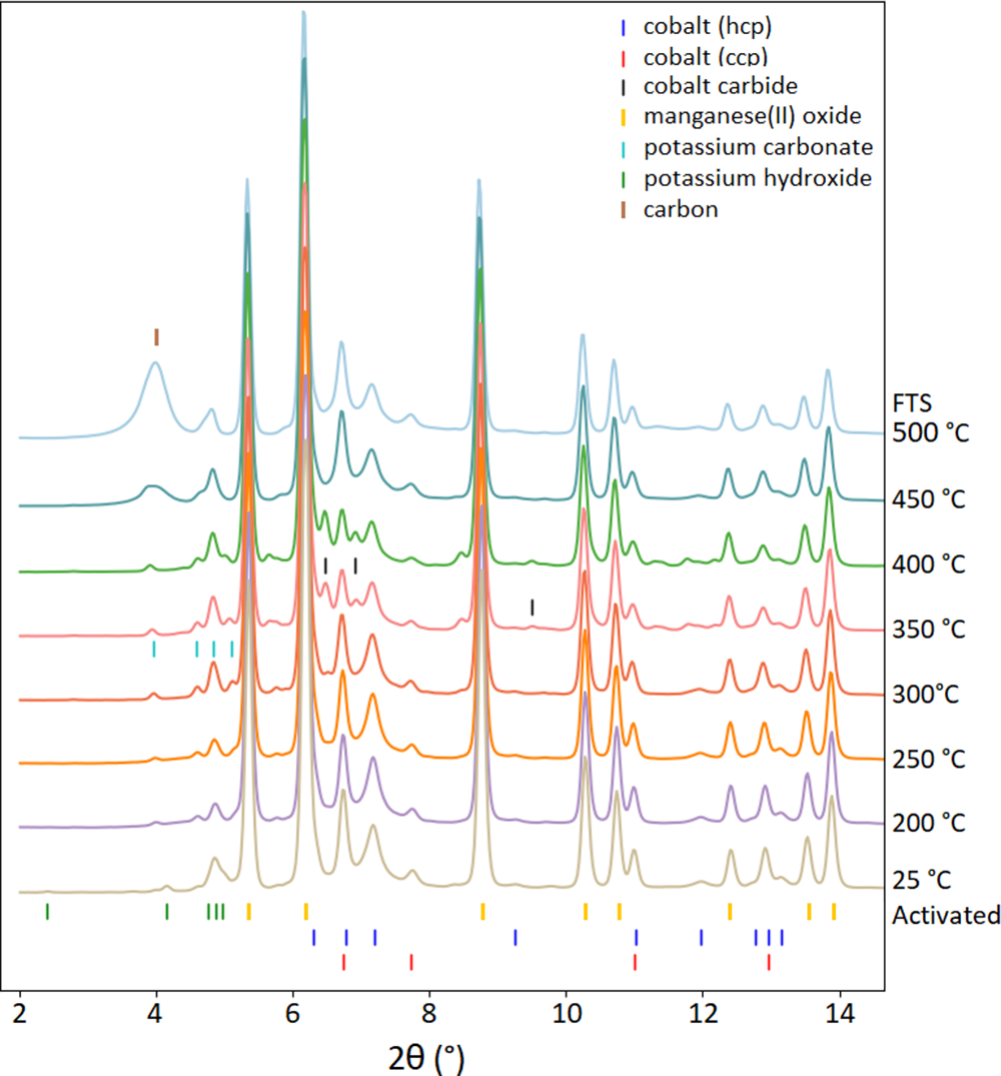
Evolution of the diffractograms of the activated Co-doped birnessite sample during the FTS catalysis process with increasing temperature.

In the case of the cobalt-doped birnessite precatalyst, the material converted to a mixture of manganosite (MnO), potassium hydroxide (KOH), and elemental cobalt upon activation. A plot of in situ X-ray powder diffractograms obtained for the catalyst phase evolution during catalysis at increasing temperatures can be seen in Figure 12. Evolution of phase abundances is shown in Figures S11, S19, and Table S3. Similar to the LiCoO_2_ experiment, both metal Co phases appeared after the activation. Also formed were MnO and KOH, with degradation production from the birnessite precatalyst. The total concentration of both Co metal phases was about 20 wt %, consistent with the cobalt composition used in the synthesis of the doped birnessite precatalyst. KOH was quickly transformed to K_2_CO_3_ upon heating to 200 °C. The metal Co phases were partially carbonized to Co_2_C starting at 300 °C. The carbide abundance peaked at 400 °C, reached 31 wt % of all-cobalt-related phases, and disappeared at 450 °C. The nano carbon phase appeared at 450 °C, having an estimated size of 4 nm, same as the carbon phase observed in the LiCoO_2_ experiment. It is worth pointing out that in both experiments, carbon formation occurred at the same temperature, and the Co_2_C phase transformed back to metal Co. In the case of the birnessite sample, this temperature was 450 °C, while it was 400 °C in the LiCoO_2_ experiment.

**Figure 13:**
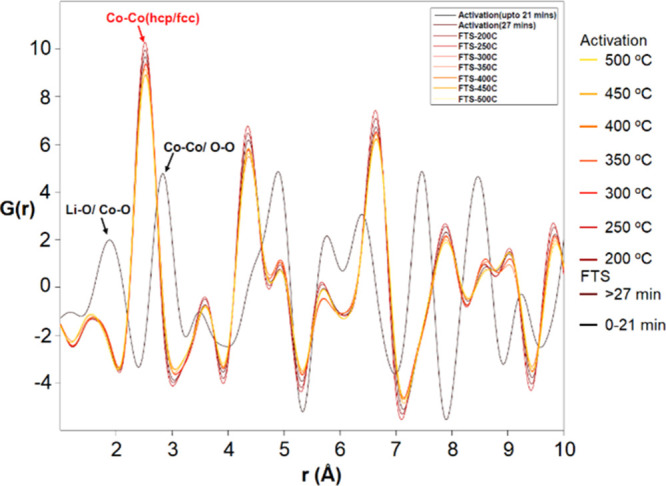
PDF: G(r) vs distance as a function of reaction progress for the LiCoO_2_ precatalyst.

The pair distribution function G(r) reduced from the data set is shown in Figure 13. The radial distribution functions of the diffractograms were overlayered, and modifications in interatomic distances are visible during reduction and catalysis. During the activation phase up to 21 min, the peaks at 1.97 Å corresponding to Li–O or Co–O; 2.82 Å corresponding to Co–Co or O–O interatomic distances were observed. The peaks at 1.97 and 2.82 Å disappeared at 27 min of reduction process and evolved into a peak at 2.52 Å corresponding to that of metallic Co (hcp/fcc) phases.

**Figure 14:**
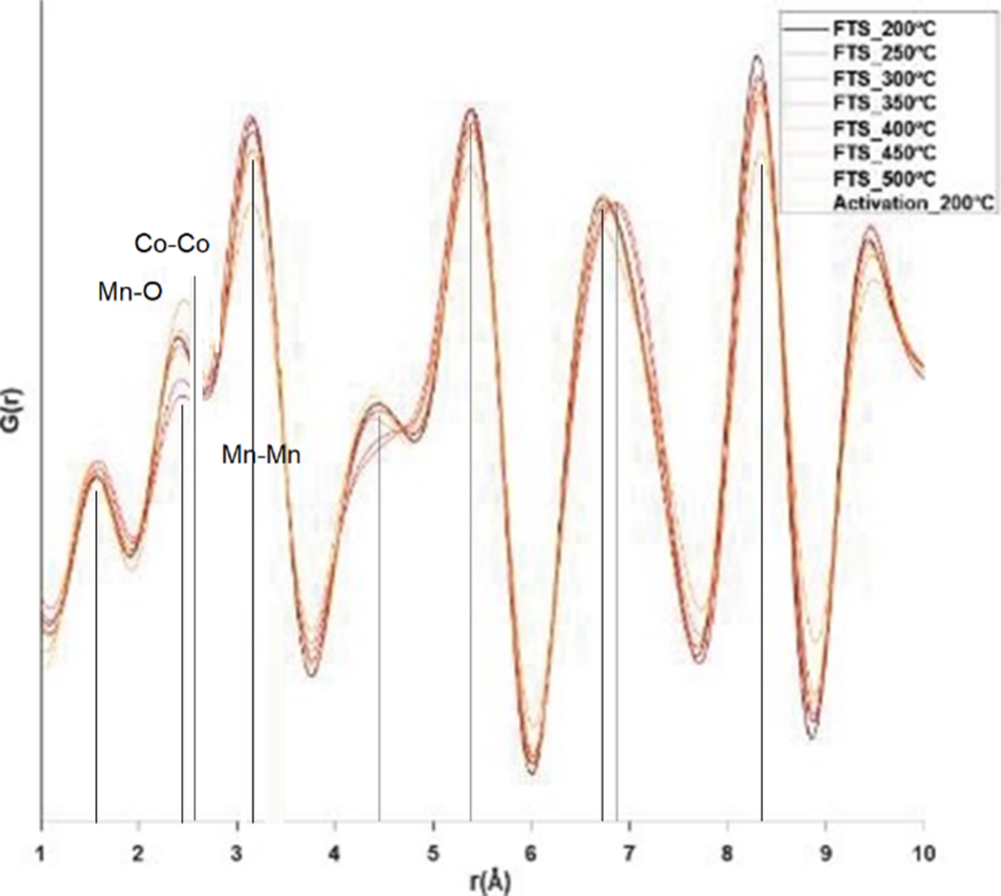
PDF: G(r) shows the reaction progress for the cobalt-doped birnessite precatalyst. The appearance of the metallic cobalt catalyst can be observed by the rightward shift of the maximum near 2.5. This peak corresponds to a combination of manganosite Mn–O and hcp cobalt Co–Co bonds.

The pair distribution function G(r) refined for the mixed Mn–Co catalyst data set is shown in Figure 14. The radial distribution functions of the diffractograms were overlaid for the reduction and catalysis stages of the process. The peak at 2.357 Å corresponds to Mn–O distances and shifts to higher distance following activation as Co–Co contacts in the cobalt hcp nanocatalyst appear. The peak at 3.157 Å corresponds to the Mn–Mn distance in the manganosite phase.

## Discussion

LiCoO_2_ (lithium cobaltite) and cobalt-doped birnessite were synthesized, characterized, and their suitability as precatalysts in Fischer–Tropsch synthesis were evaluated. The catalysts were first activated by treatment with hydrogen gas at 200 °C. The activation process reduced cobalt to metallic cobalt phases (fcc and hcp) and manganese to manganosite, as determined by in situ X-ray powder diffraction (Figures 11 and 12). After activation, the catalyst was exposed to the syngas mixture. The metallic cobalt phases generated on the surface of the reduced catalyst are hypothesized to catalyze the FTS. The large surface area of LiCoO_2_ is presumed to facilitate the synthesis of large surface area cobalt particles that ensure sufficient adsorption of syngas for CO activation. The fitted particle sizes of 20 nm for the Co metal phases are consistent with this. For the all-cobalt system, the product analysis suggested that the reaction exhibited an overall high %CO conversion (average 70.5% CO conversion) comparable to previously studied Co-based catalysts such as MnO_*x*_/Co, TiO_2_/Co, and CNT/Co (CNT, carbon nanotubes).^19,22^ Products up to C_7_ were identified with paraffins as dominant products, compared to olefins. The total product selectivity generally increased up to 300 °C but decreased beyond this temperature. This shift in the product selectivity and concurrent rise in %CO_2_ selectivity can be attributed to the domination of the water gas shift reaction at higher temperatures. In the case of the cobalt catalyst on the manganosite support, conversion generally increased with increasing temperature, and selectivity for long-chain hydrocarbons was lower and decreased further with increasing temperature. While this phenomenon is well-established for cobalt-based FTS catalysts,^23^ the effect is diminished in our materials. This is likely due to the inclusion of other phases in the postactivation catalyst, including lithium salts that form following activation of the LiCoO_2_ precatalyst (Figure 11) and manganese(II) oxides (Figure 12). Consistent with this hypothesis, the cobalt-doped birnessite (which has the higher proportion of noncobalt mass) retains higher selectivity for longer chains at higher temperature. We hypothesize that this is due to the Mn-based phase preventing aggregation and sintering of the cobalt catalyst at about 400 °C, which facilitates one-carbon chemistry and hinders the formation of longer carbon chains.^23^ In general, selectivity for olefinic species was good but much greater for the MnO-supported catalyst, with olefinic excess reaching as much as 20x. Above 400 °C, however, there is a sharp decrease in the formation of longer carbon chains and a decrease in the olefin:paraffin ratio. This may be attributed to the sintering of catalytic particles at high temperature, which is less pronounced than pure Co-FTS catalysts, likely due to the role of additional Li- and Mn-derived phases (detected in Figures 11 and 12) that may physically prevent sintering of the active cobalt particles below 400 °C. However, the effect is much less pronounced for the Mn–O-containing catalyst, which shows a decrease in the chain length and an overall decrease in the olefin:paraffin ratio, but continued generation of ethylene above 400 °C (Figure 10). In contrast, in the Li–Co system, all olefinic production is shut down above 400 °C (Figure 8). The prevention of sintering of nanocatalysts by nanoconfinement in metal oxides frameworks is well-known,^24^ and cobalt catalysts suspended in zeolytes gave similar effects.^25^ These observed loss of long-chain selectivity, but persistence of ethylene production for the MnO-supported catalyst is consistent with an increased rate of β-hydride elimination as a reaction termination step for the manganese-supported catalyst. The dispersal of the catalyst in the matrix may also prevent the readsorption of olefinic products, facilitating termination of the catalysis at shorter olefinic products.

In situ studies revealed that Co(hcp) and Co(fcc) formed during the activation step of the FTS reaction. Previous studies suggested that the Co(hcp) crystal phase is more likely to have higher FTS activity as compared to that of the Co(fcc) phase.^26^ Zhang et al. suggested CHO-insertion and a carbide mechanism for the initial formation and growth of carbon chain over Co(hcp) and Co(fcc) facets. Previously reported DFT calculations reveal the higher CO dissociation rate on most of the Co(hcp) facets compared to that of Co(fcc).^27^ Additionally, the computational results suggested that direct CO dissociation (CO* + H* → C* + O* + H*) is preferred on Co(hcp). While the hydrogen-assisted CO dissociation (CO* + H* → CHO* → CH* + O*) is thermodynamically favored on Co(fcc). The potential energy of the direct pathway on Co(hcp) is lower than that of hydrogen-assisted dissociation on Co(fcc), indicating the higher activity of Co(hcp). It was also reported that the carbide mechanism on Co preferably suggested higher selectivity of C_2_ hydrocarbons as compared to methane.^20^ The amount of the Co(hcp) phase was approximately 4 times as compared to that of the Co(fcc) phase. The Co(hcp) phase fraction decreased with increasing temperature, which is concurrent with the decrease in the total product selectivity. The product analysis suggested that the C_2_ selectivity was indeed higher as compared to that of methane, which indicated that the carbide mechanism was the plausible mechanism of the FTS reaction. The accumulation of detectable amounts of cobalt carbide from in situ PXRD (Figures 11 and 12) is consistent with this mechanism.

The activity and selectivity of the Fischer–Tropsch synthesis are size-dependent.^28,29^ Iglesia reported that the turnover frequency (TOF) of the FTS process is insensitive to the cobalt particle size over a range of 10–200 nm.^30^ However, the methane selectivity dramatically increases as the cobalt particle size decreases below 10 nm, and the TOF decreases within smaller nanoparticles. de Jong et al. reported that the TOF of CO hydrogenation remains almost unchanged when particle sizes are larger than 6 or 8 nm but decreases with smaller particle sizes (<6 nm).^29^ As the particle size decreases, the amount of exposed low-coordinated cobalt atoms on the catalyst surface increases, increasing the rate of reaction of cobalt with H_2_O to form Co–O bonds and thereby enhancing methane selectivity. The larger metallic cobalt particles have more stable sizes because of more difficult oxidation of surface metallic cobalt sites, which coincides with low methane selectivity at lower catalysis temperatures.

In the FTS reaction, olefins are formed by hydrogen abstraction. The olefins can leave the reaction zone as product, be readsorbed on the Co surface, or hydrogenated to secondary paraffins.^30^ The extent of readsorption depends on the degree of mass transfer restriction on the product removal, which, in turn, depends on the catalyst pore size and active site density. However, Shi and Davis studied the O/P ratio by switching H_2_ and D_2_.^31^ They concluded that α-olefin readsorption does not have a major impact on the product distribution. The Fischer–Tropsch synthesis involves a chain growing process on the Co surface leading to methane, olefins, and paraffins. The relationship between the O/P ratio and particle size is due to the changes in hydrogenation activity on the catalyst. For small particles, the support contributes to increased hydrogenation activity either directly or indirectly through the Co particle. A relative increase in termination by hydrogen abstraction for larger particles tends to work in parallel with the facilitation of CO activation and creation of CH_*x*_ monomers up to 8–10 nm. For even larger particles, the decline in selectivity is not easily understood.^32^

Co-doped birnessite was also prepared as a precatalyst for the Fischer–Tropsch synthesis. The large surface area of 117.6 m^2^/g is presumed to facilitate the sufficient adsorption of syngas for CO activation. Light olefins and paraffins (up to C4) were selectively synthesized. Methane selectivity was low up to 350 °C (2.86%C) but increased to ∼10% at 400–450 °C and 48.35% at 500 °C. The total product selectivity at 200–300 °C was 75.9% (C2–C4), which increased to 85.50% at 350 °C, but beyond this temperature, the reaction was less selective for longer-chain products, and C3–C4 products were not observed above 350 °C, with further decreased product selectivity to an average of 40% of C2 at 400–500 °C. %CO_2_ selectivity was low (8.9%C average) up to 350 °C, which increased to an average of 37.1%C at 400–500 °C. This shift in the product selectivity and concurrent rise in %CO_2_ selectivity can be attributed to the enhanced water gas shift (WGS) reaction at higher temperatures.

Product analysis suggested that MnO/Co displayed higher selectivity toward lower olefins and low selectivity toward undesired methane and lower paraffins (Table 4). The mechanistic steps germane to the FTS catalysis include chain growth, chain branching, primary olefin/paraffin formation, and olefin secondary reactions such as secondary hydrogenation and isomerization.^19^ From Figure 10 and Table 4, MnO/Co produced significantly more primary olefins than linear paraffins for each carbon number. This suggests that β-H elimination was the dominant termination pathway, and secondary hydrogenation of olefins was also suppressed.^19^

**Table 4:** Olefin/Paraffin Ratio as a Function of Temperature

carbon count (*n_i_*)	olefin to paraffin (o/p) ratios						
	200 °C	250 °C	300 °C	350 °C	400 °C	450 °C	500 °C
2	2.84	3.89	1.91	2.14	0.093	8.13	6.14
3	6.37	8.17	8.67	7.9			
4	21.87	16.6	19.16	16.6			

In situ studies revealed that nanoconfined Co(hcp) and MnO (ccp) phases evolved during the activation step of the FTS reaction. Previous studies suggested that MnO served as a support for the metallic Co nanoparticles, thereby ensuring a good dispersion and stabilization of these nanoparticles.^8^ MnO is also known to act as an electronic and structural promoter, and the promoting effects of MnO are strongly dependent on its location and amount. Morales et al. showed that CO preferentially bonded linearly to surface metal sites when MnO loading was increased.^33^ However, in the mentioned literature, the MnO_*x*_ loading was much lower than the Co loading, and the promoting effects of MnO are not yet clear.

## Conclusions

The activity and in situ speciation of FTS catalysts derived from cobalt-containing layered precatalyst materials were explored in the present work. The in situ XRD characterization provided useful insights into the evolution of catalytically active crystalline phases for the FTS reaction that were otherwise challenging to detect using conventional ex situ methods. The results suggested that cobalt was reduced to Co(hcp) and Co(fcc) nanoparticles, providing a high surface area medium for the reduced metallic cobalt particles to catalyze CO hydrogenation. In the presence of manganese precursors, the catalyst evolves into a nanocobalt catalyst supported on a manganosite matrix. The alkali metal promoters have been reported to improve chain growth probability and olefin-to-paraffin ratio by increasing the electron density on the catalyst surface in previous FTS studies. In our work, the catalysts generated paraffins up to seven carbons in length, and under moderate temperature (350 °C), five-carbon chains were the major product. The segregation of the cobalt nanocatalyst within a manganosite support by the use of a cobalt-doped manganese oxide precatalyst resulted in similar FTS chemistry, but a change in selectivity wherein shorter-chain alkanes were formed in greater amounts but with a greater selectivity for olefins (up to about 20× excess olefins for the Mn–Co catalyst, whereas the maximum olefinic excess was only about 2× for the all-cobalt catalyst). The results show that mixed composition precatalysts can be used to prepare supported catalysts in situ for the tuning of selectivities based on precatalyst composition. The role of Lewis cations K^+^, Li^+^, and the supporting MnO on catalysis is of interest for future work on these systems.

## Supporting Information Available

Additional figures including diagrams of experimental apparatus, additional EDS and XPS spectra, BET data, ASF plots, and plots of crystal phase, size, and strain from in situ PXRD; additional tables of experimental parameters for GC and PXRD (PDF)

## Abbreviations

GtL: gas-to-liquid
CtL: coal-to-liquid
BtL: biomass-to-liquid
FTS: Fischer–Tropsch synthesis
TEM: transmission electron microscopy
SEM-EDS: scanning electron microscopy-energy dispersive spectroscopy
XPS: X-ray photoelectron spectroscopy
VSSA: volume-specific surface area
BET: Brunauer–Emmett–Teller analysis
FID: flame ionization detector
PXRD: powder X-ray diffraction
APS: Advanced Photon Source
ANL: Argonne National Laboratory
COD: Crystallography Open Database
WGS: water–gas shift
hcp: hexagonal close-packed
ccp: cubic close-packed
PDF: pair distribution function
CNT: carbon nanotube
