# Supplementary material for: Hydrocarbon Formation from Syngas with In-Operando Monitoring of Cobalt- and Manganese-Based (pre)Catalysts Using X-ray Diffraction
Source: ACS Omega. 2024 Jun 25;9(27):29917–27. doi: 10.1021/acsomega.4c04553 (PMC11238217; doi:10.1021/acsomega.4c04553)
Supplement: Supplementary file 1 — ao4c04553_si_001.pdf [file ao4c04553_si_001.pdf]

Supporting Information for

Hydrocarbon formation from syngas with in-operando monitoring of cobalt- and manganese-based (pre)catalysts using X-ray diffraction.

Ravneet K. Bhullar,[a†] Wenqian Xu,[b] Michael J. Zdilla.[a*]

Contents

Apparatus diagrams. 3

X-ray spectroscopy 4

BET 6

Anderson-Schulz-Flory Analysis 7

Pair Distribution Function Data. 17

ICP-OES 18

Additional Tables 19

Apparatus diagrams.

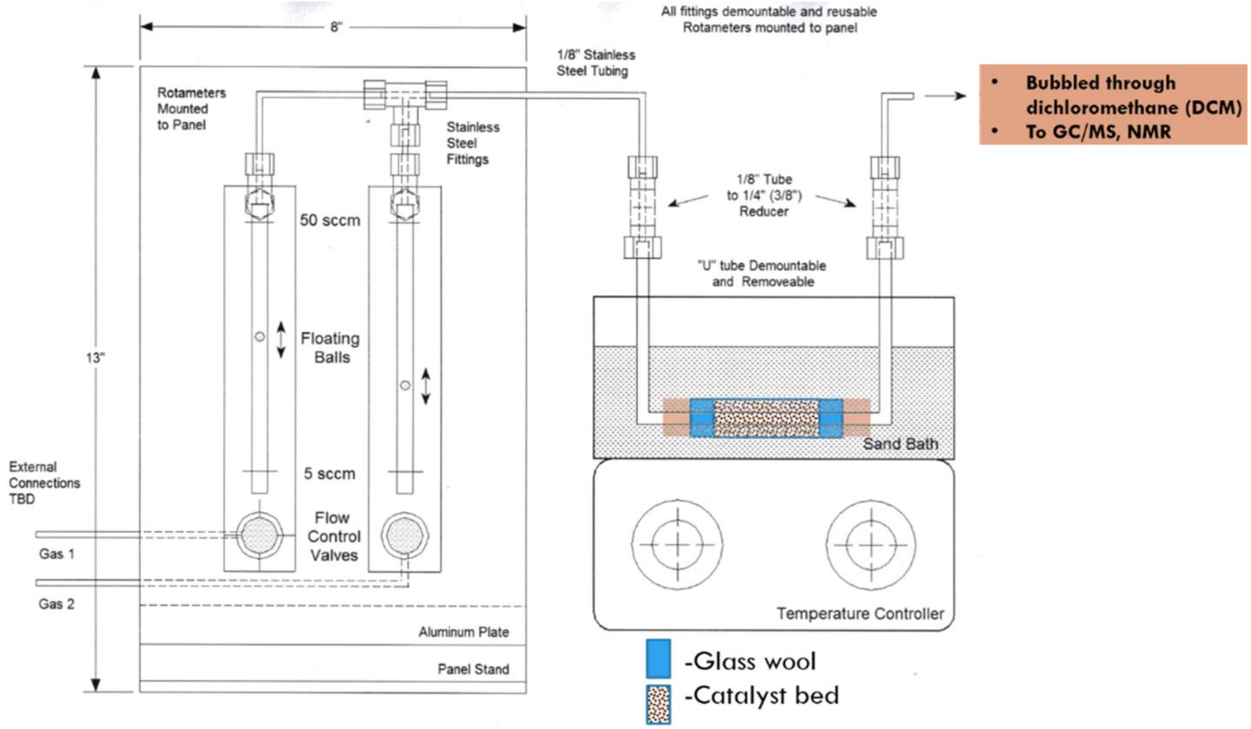

Figure S1. Custom stainless steel reactor apparatus.

X-ray spectroscopy

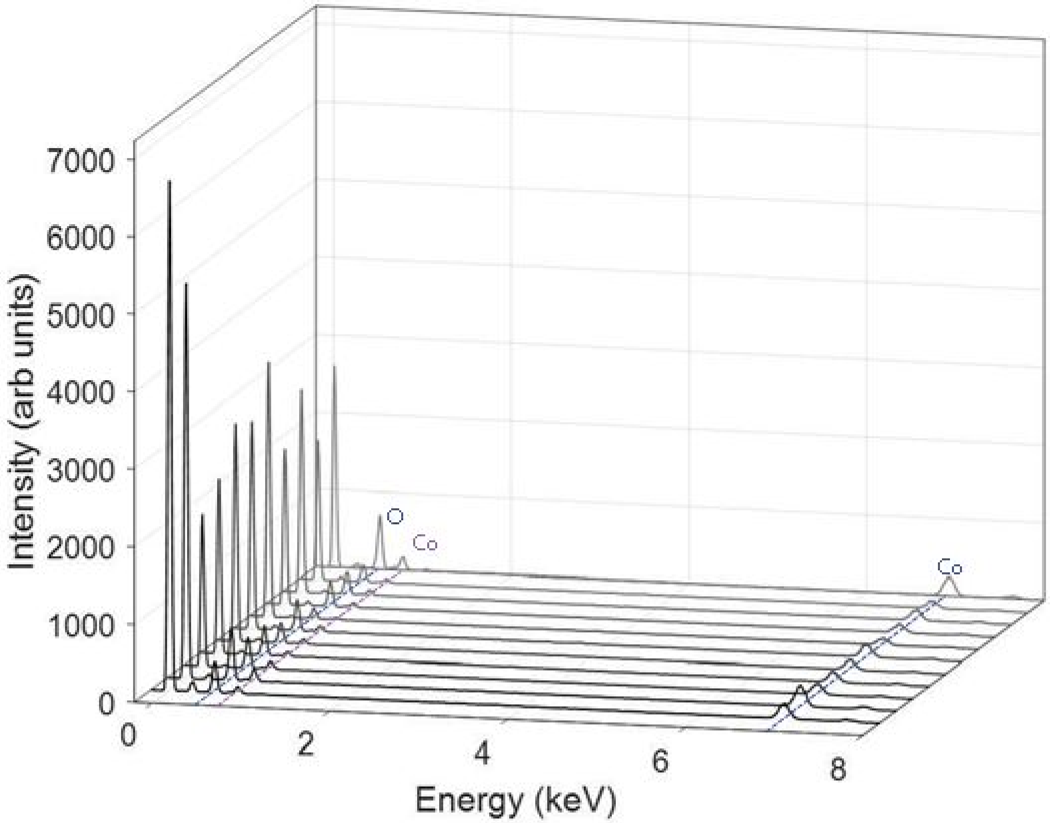

Figure S2. EDS spectra of $LiCoO_2$ sampled at multiple crystals.

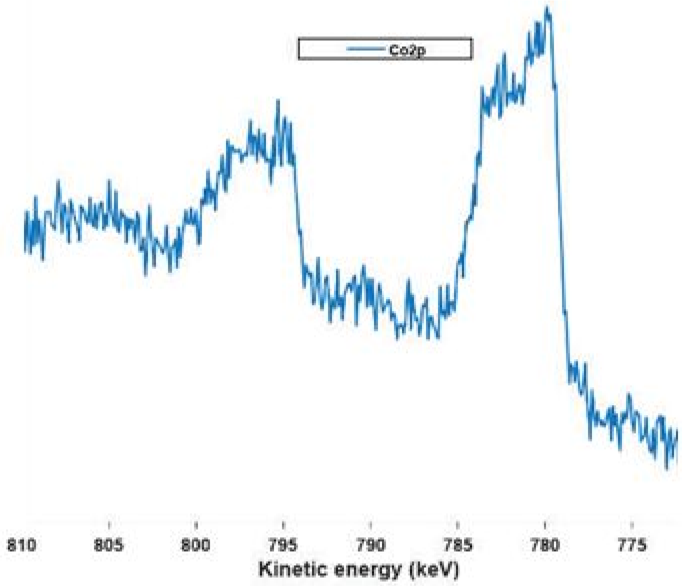

Figure S3. Cobalt region of the XPS of $LiCoO_2$

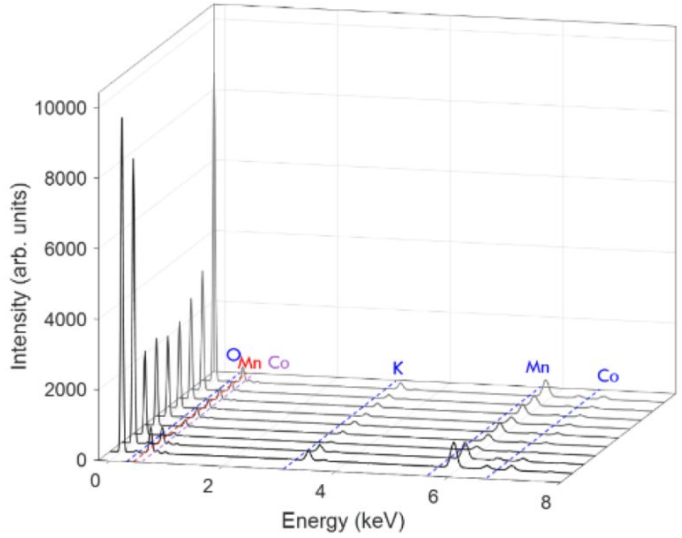

Element	Average Atomic %
Oxygen	52.3
Manganese	31.7
Cobalt	9.5
Potassium	6.5

Figure S4. EDS spectra of cobalt-doped birnessite sampled at multiple crystals.

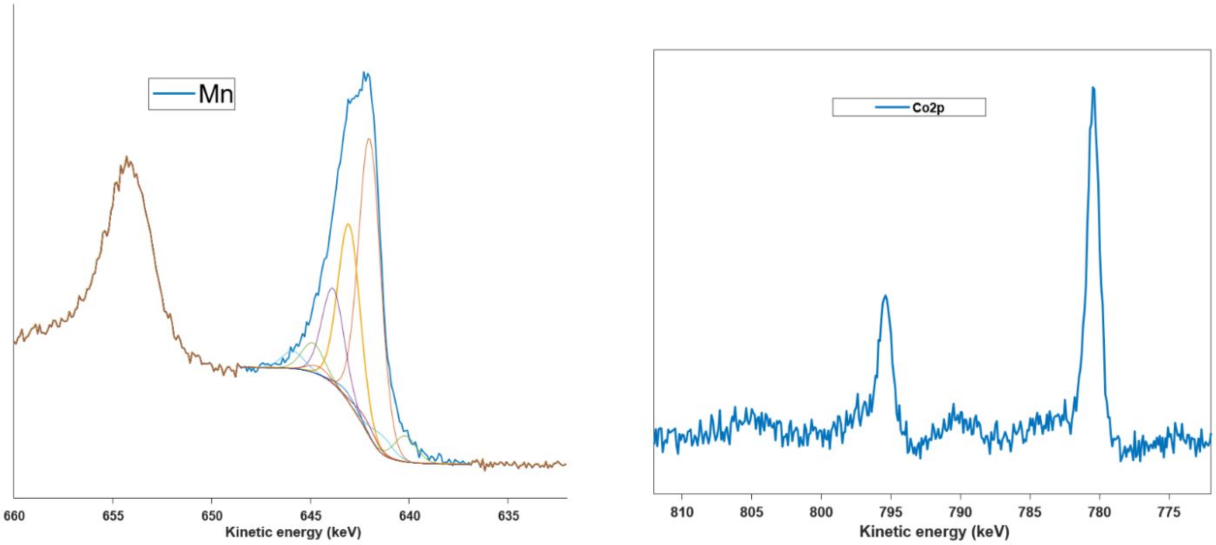

Figure S5. Mn and Co 2p XPS of cobalt-doped birnessite.[10]

BET

The BET adsorption isotherm in Figure S4 was identified as type II isotherm, a characteristic of a non-porous or microporous solid. The shape was the result of unrestricted monolayer-multilayer adsorption up to capillary condensation. The total surface area of the sample material was 348 m^2/g. The volume specific surface area of 72.75 m^2/cm^3 indicated that the sample can be identified as nanomaterial according to the official EC recommendation as the value of VSSA was higher than 60 m^2cm^{-3}.

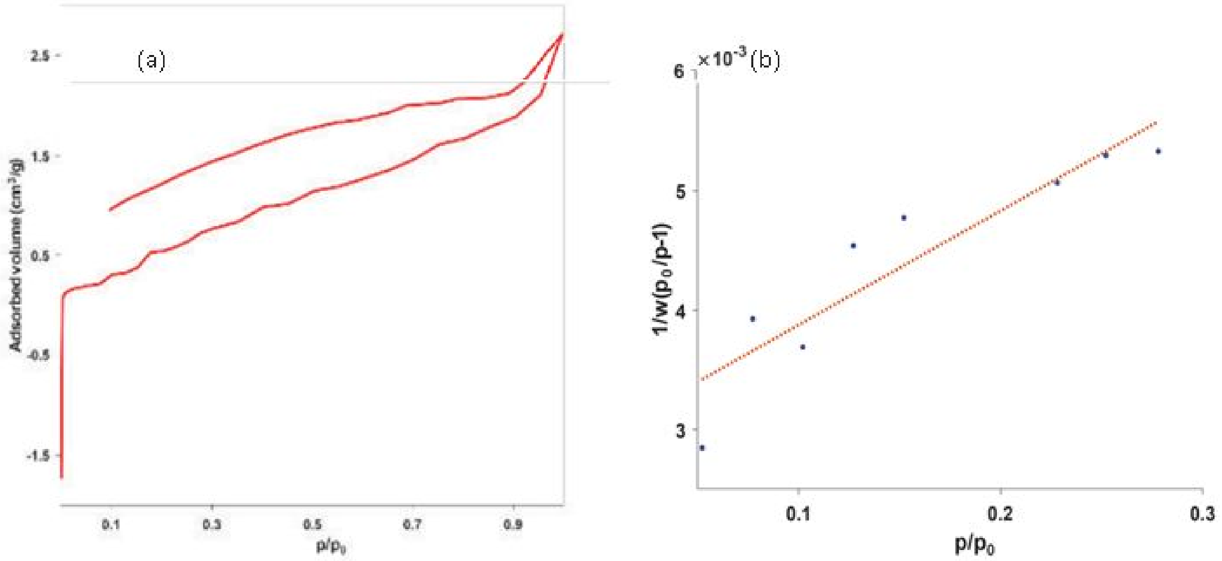

Figure S6. (a) BET isotherm (b) multipoint BET for $LiCoO_2$ for the surface area analysis.

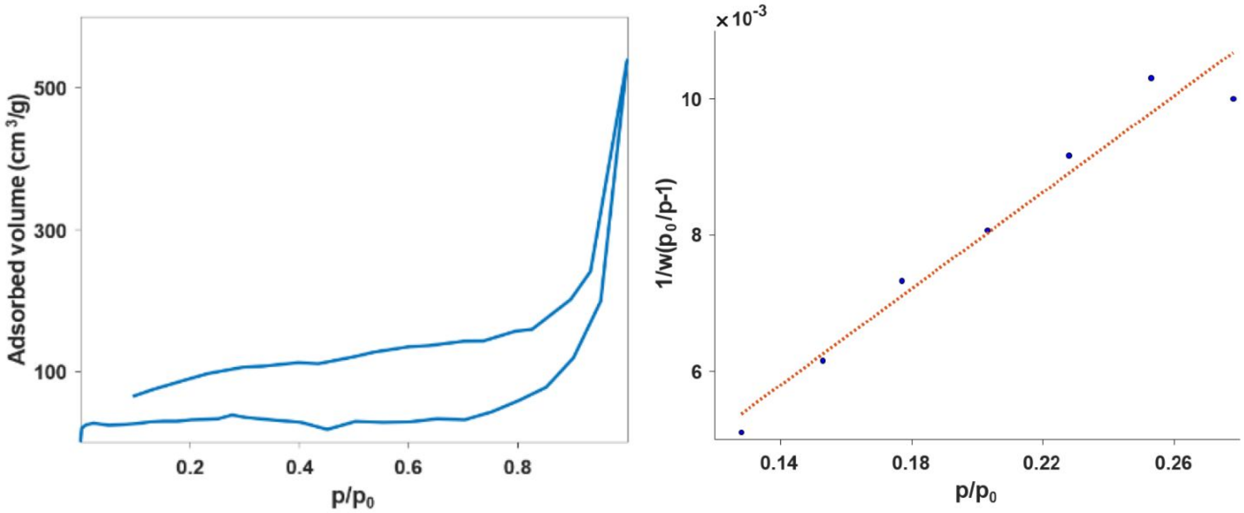

Figure S7. (a) BET isotherm (b) multipoint BET for cobalt-doped birnessite for the surface area analysis.

Anderson-Schulz-Flory Analysis

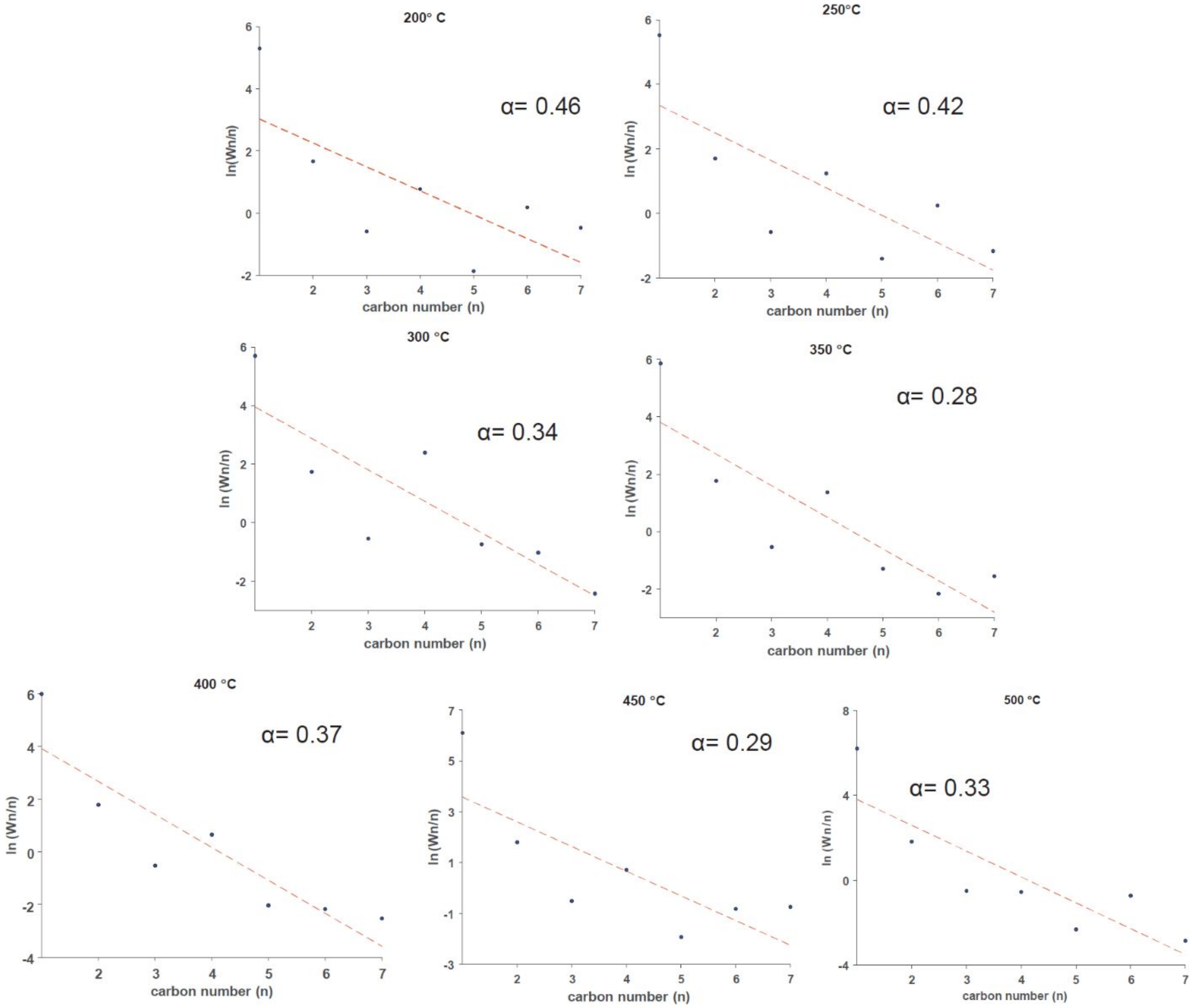

Figure S8. Anderson-Schulz-Flory plots for catalysis at (a) 200°C (b) 250°C (c) 300°C (d) 350°C (e) 400°C (f) 450°C (g) 500°C

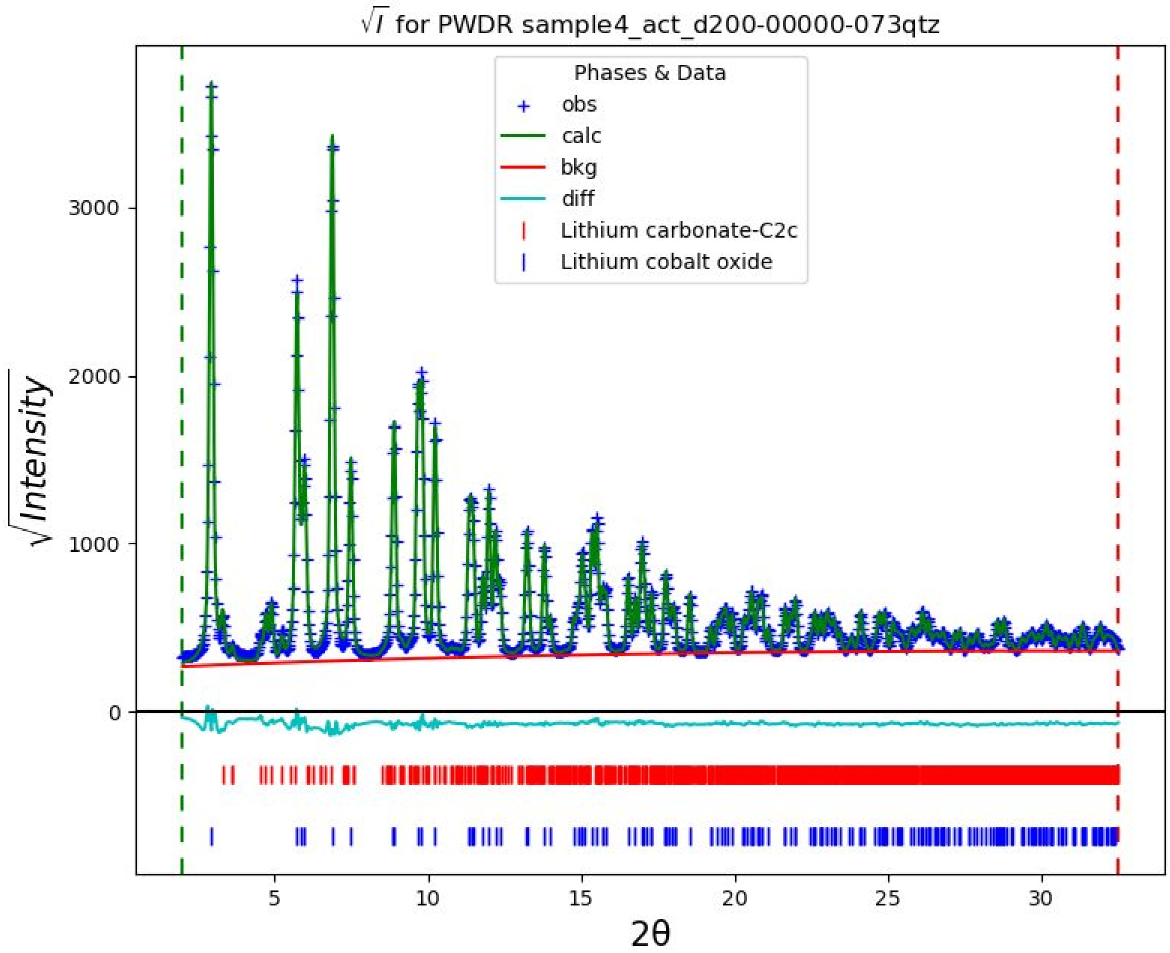

Figure S9. Refinement plot of the all-cobalt catalyst before the activation. The result shows the sample contains 89.2 wt% of $LiCoO_2$ and 10.8 wt% of Li_2CO_3. R_{wp} = 3.8%. λ = 0.24105 Å. Background scattering from the fused quartz capillary has been subtracted.

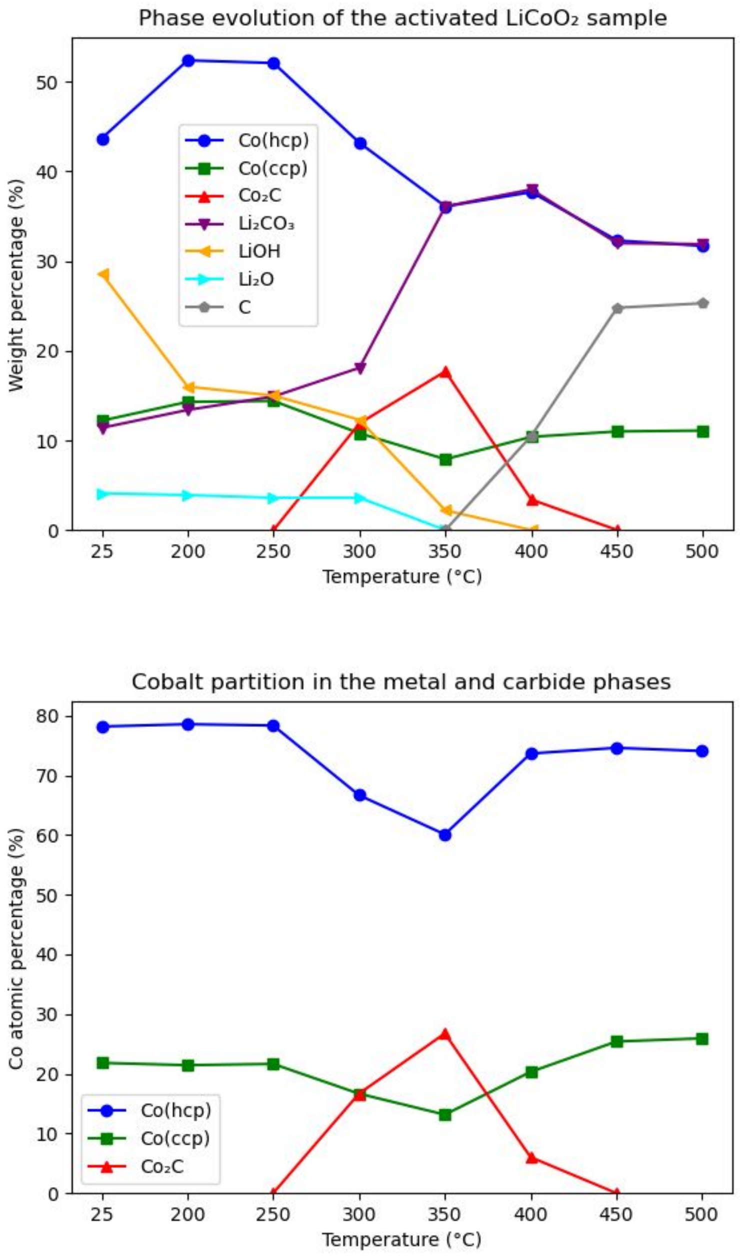

Figure S10. Evolution of phases and their abundances in the activated all-cobalt catalyst sample in the FTS catalysis process with increasing temperature (top panel), and the evolution of cobalt-bearing phases and their relative abundance (bottom panel).

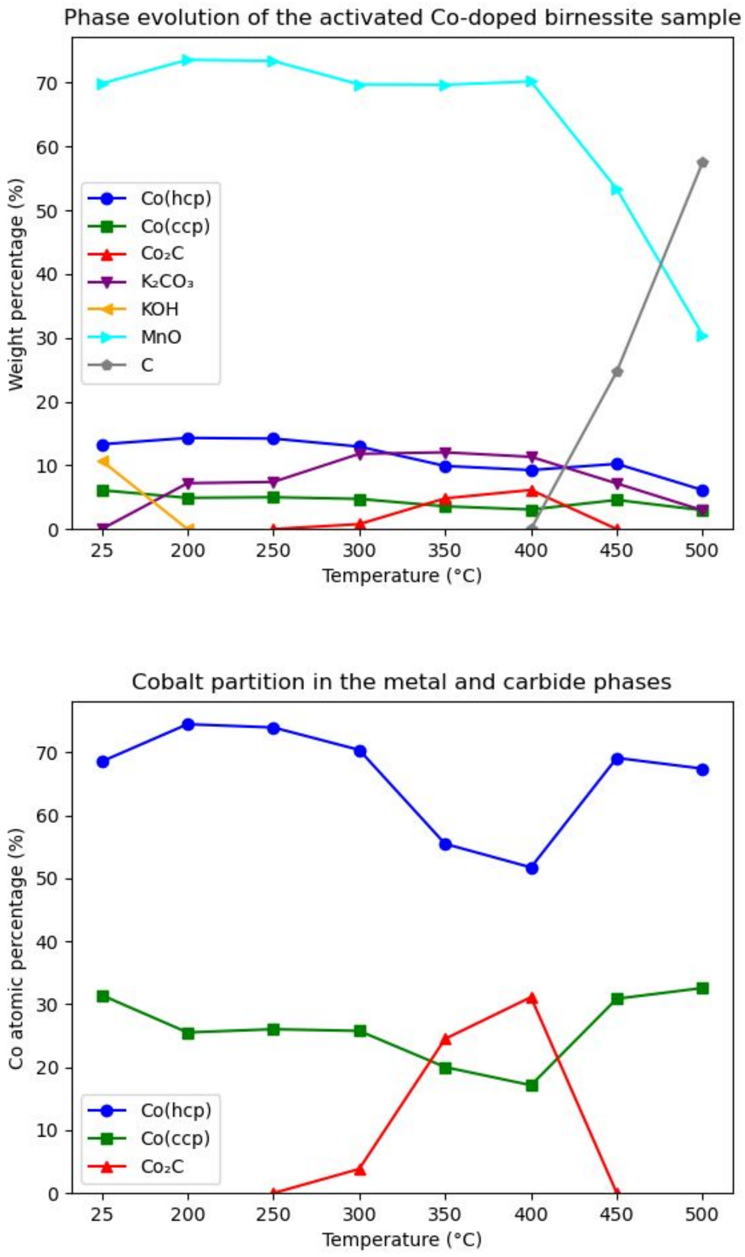

Figure S11. Evolution of phases and their abundances in the activated Co-doped birnessite catalyst sample in the FTS catalysis process with increasing temperature (top panel), and the evolution of cobalt-bearing phases and their relative abundance (bottom panel).

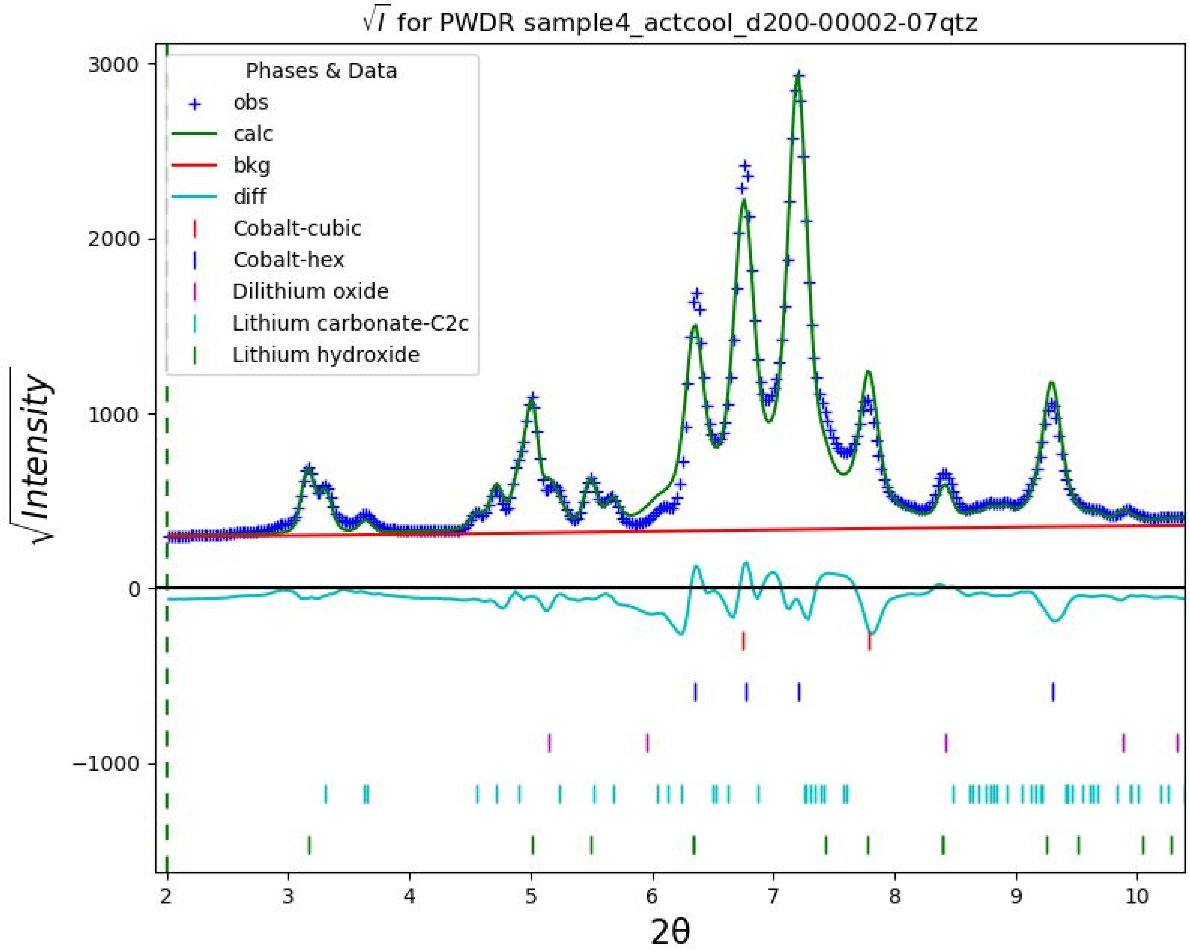

Figure S12. Refinement plot of the activated all-cobalt catalyst at 25 °C before the FTS process. The plot is only the low angle region of the whole refinement that extended to $2\theta_{max}$ of 31.8°, which equals to d_{min} of 0.44 Å. R_{wp} = 12.6%. λ = 0.24105 Å.

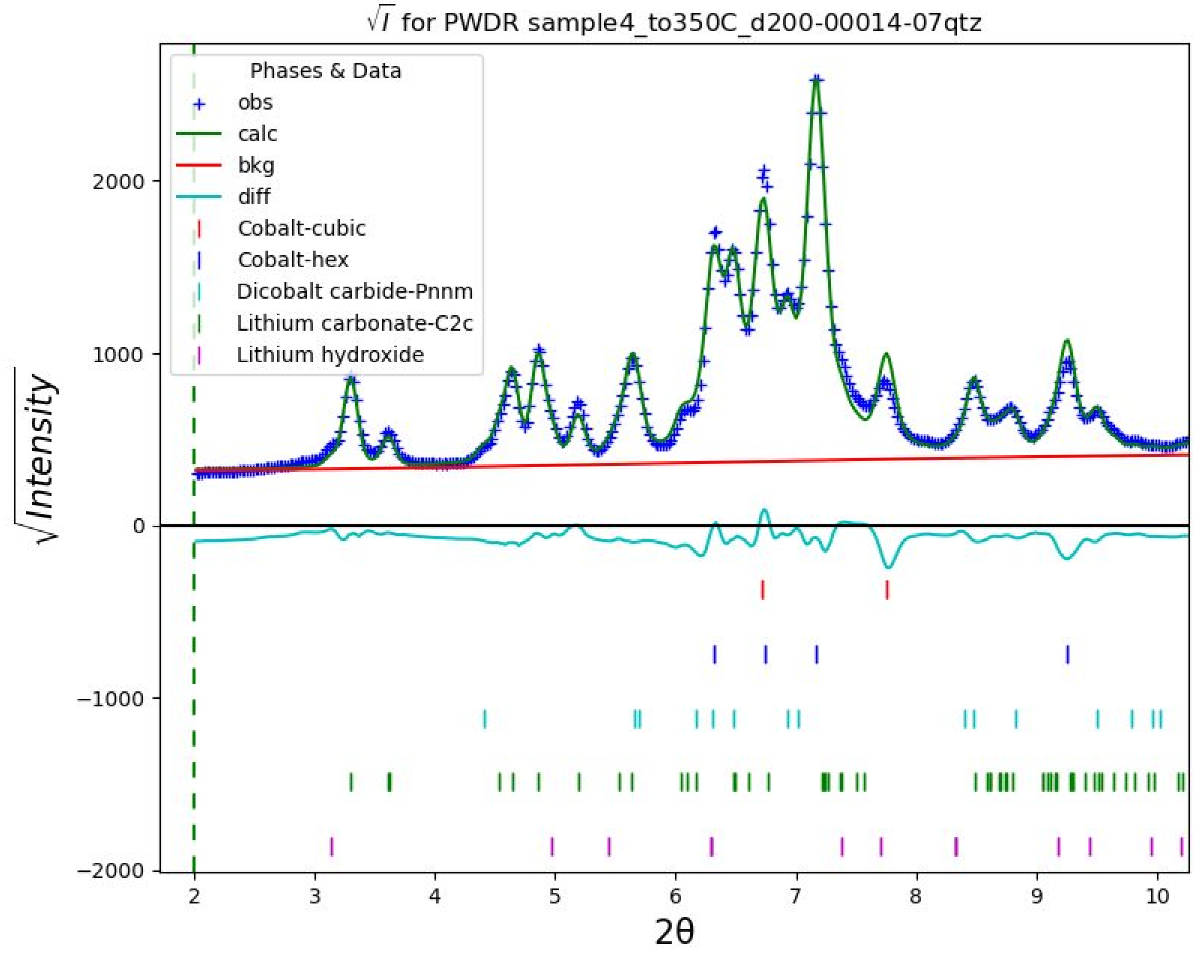

Figure S13. Refinement plot of the all-cobalt catalyst at 350 °C when the Co_2C phase peaked. The plot is only the low angle region of the whole refinement that extended to $2\theta_{max}$ of 31.8°, which equals to d_{min} of 0.44 Å. R_{wp} = 8.3%. λ = 0.24105 Å.

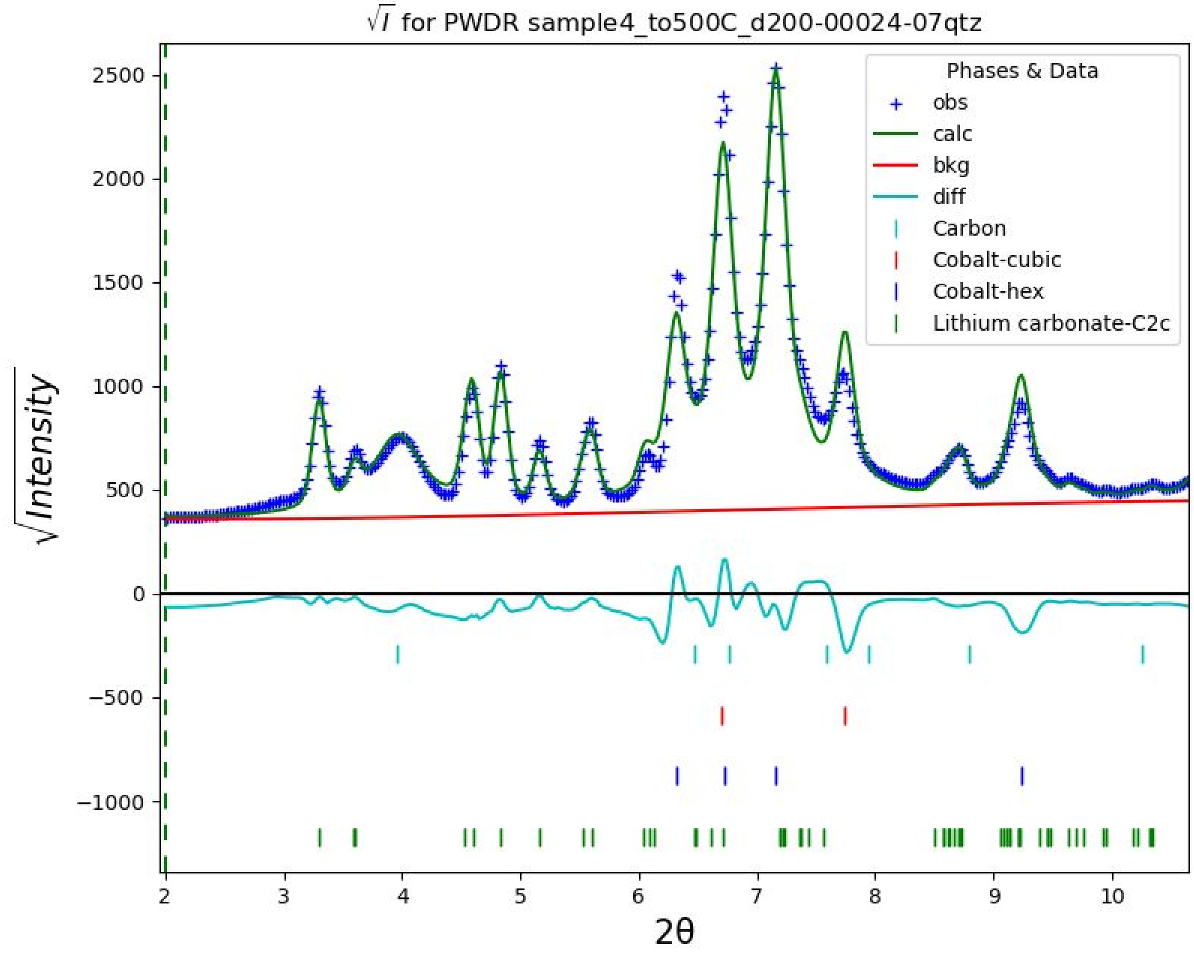

Figure S14. Refinement plot of the all-cobalt catalyst at 500 °C when carbon accumulation is clearly seen. The plot is only the low angle region of the whole refinement that extended to $2\theta_{max}$ of 31.8°, which equals to d_{min} of 0.44 Å. R_{wp} = 11.5%. λ = 0.24105 Å.

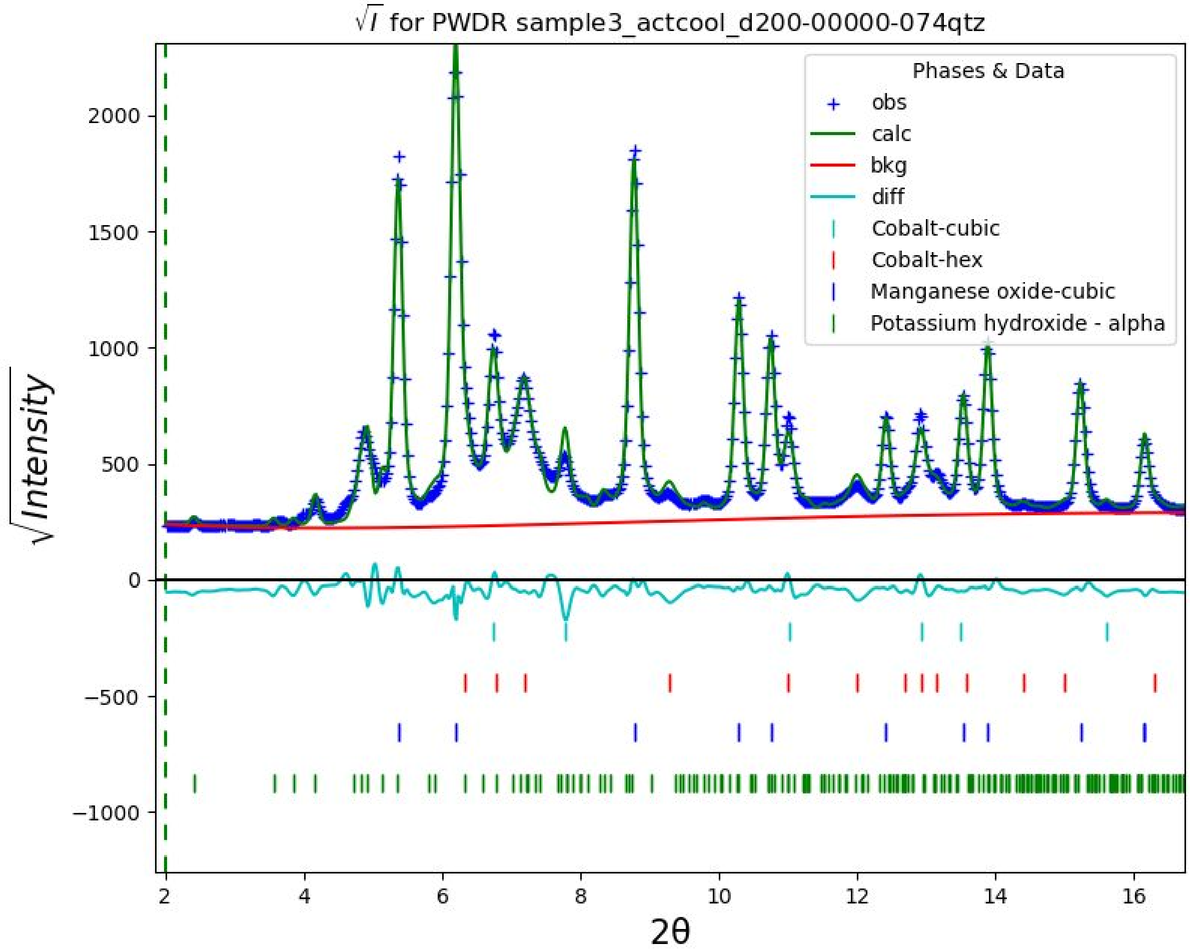

Figure S15. Refinement plot of the activated Co-doped MnO catalyst at 25 °C before the FTS process. The plot is only the low angle region of the whole refinement that extended to $2\theta_{max}$ of 33°, which equals to d_{min} of 0.42 Å. R_{wp} = 7.5%. λ = 0.24105 Å.

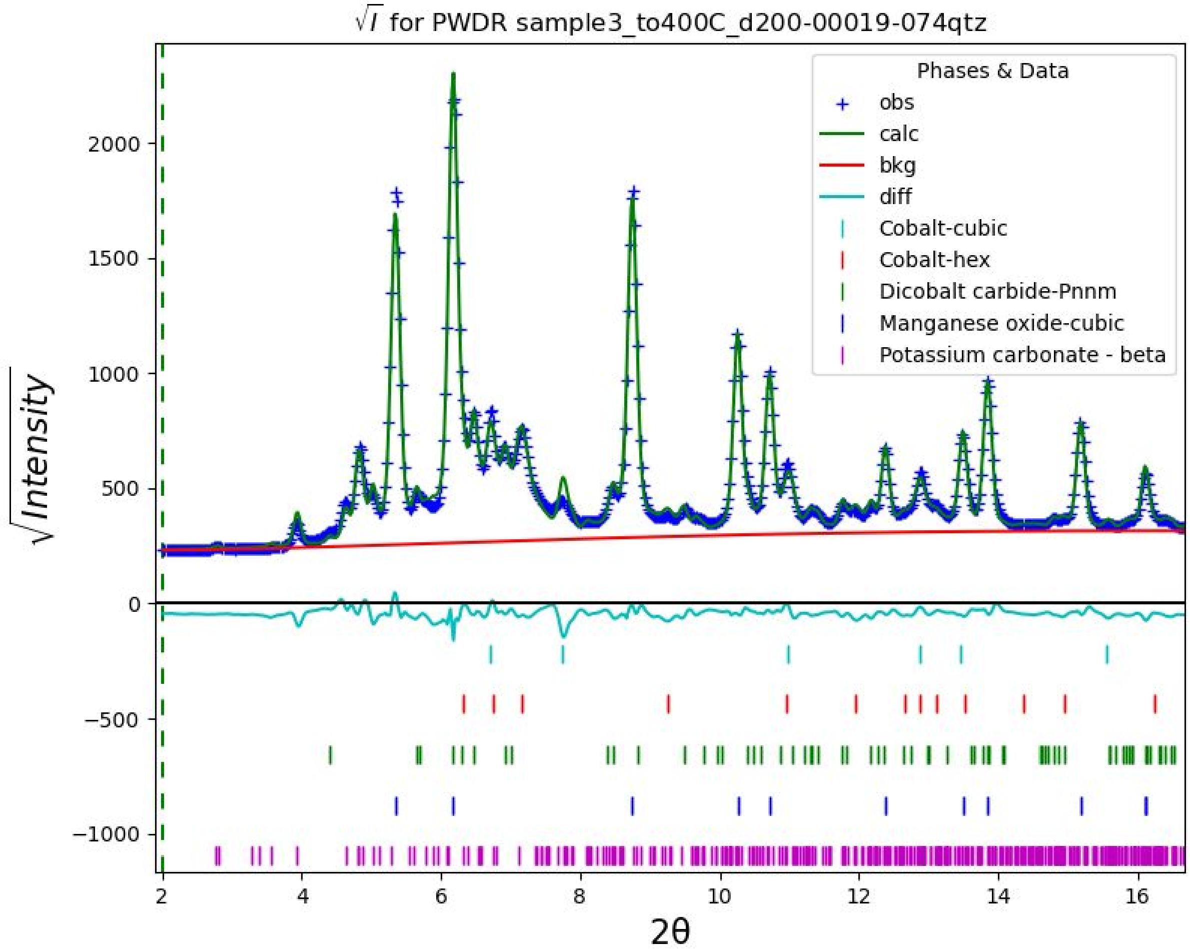

Figure S16. Refinement plot of the Co-doped MnO catalyst at 400 °C when the Co_2C phase peaked. The plot is only the low angle region of the whole refinement that extended to $2\theta_{max}$ of 33°, which equals to d_{min} of 0.42 Å. R_{wp} = 5.9%. λ = 0.24105 Å.

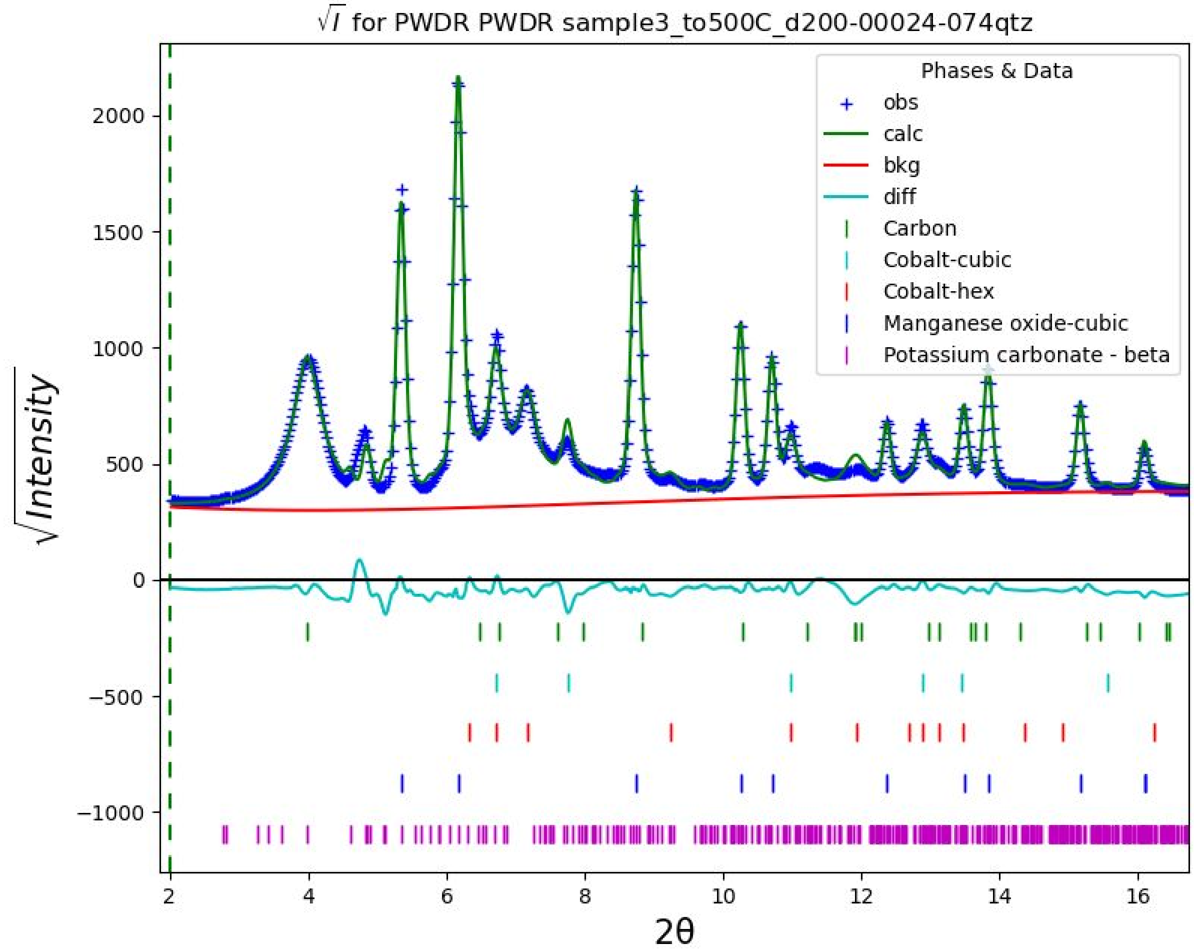

Figure **S17**. Refinement plot of the Co-doped MnO catalyst at 500 °C when carbon accumulation is clearly seen. The plot is only the low angle region of the whole refinement that extended to $2\theta_{max}$ of 33°, which equals to d_{min} of 0.42 Å. R_{wp} = 6.0%. λ = 0.24105 Å.

Pair Distribution Function Data.

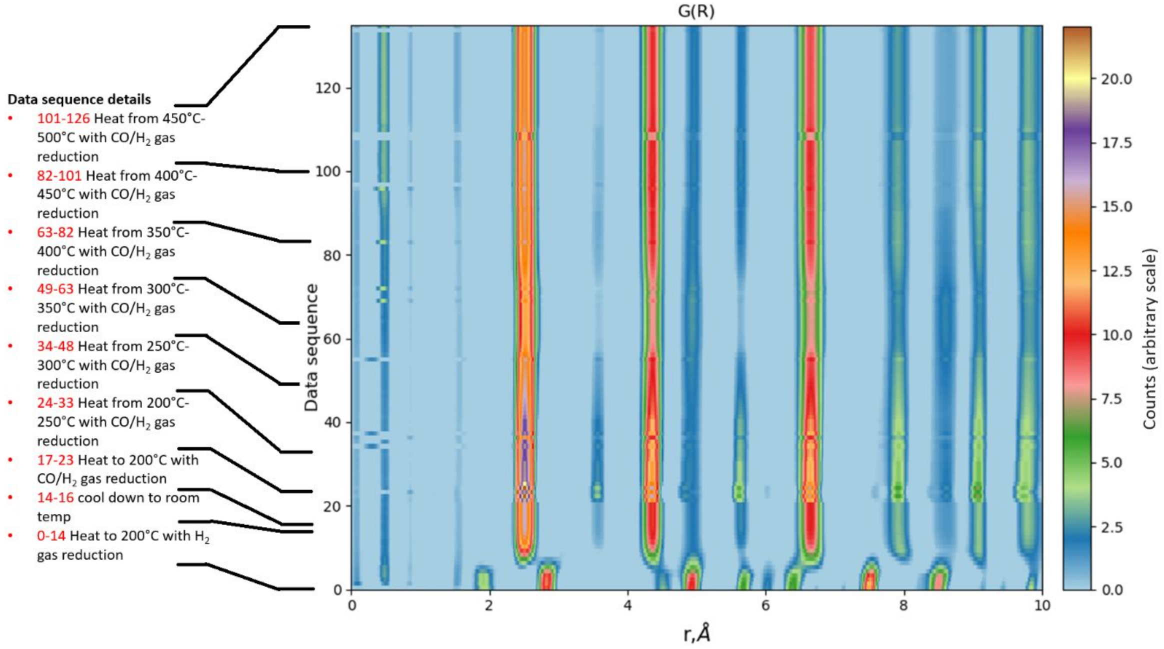

Figure S18. In-situ PDF showing phase evolution of $LiCoO_2$ precatalyst during activation and catalysis.The plot shows that activation proceeds to reduce $LiCoO_2$ to the predominantly Co phase within the first few sequences, and that the catalyst remains predominantly in this form throughout the remainder of the catalysis.

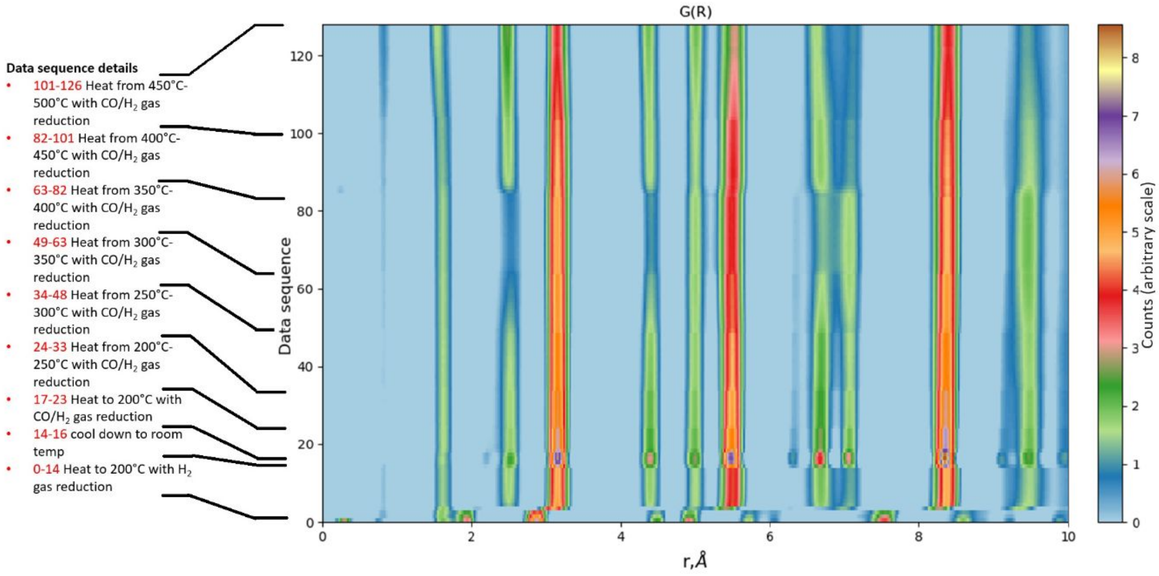

Figure S19. In-situ PDF showing phase evolution of cobalt-doped birnessite precatalyst during activation and catalysis.The plot shows that activation proceeds to reduce the precatalyst to the predominantly MnO and Co phases within the first few sequences, and that the catalyst remains predominantly in this form throughout the remainder of the catalysis.

ICP-OES

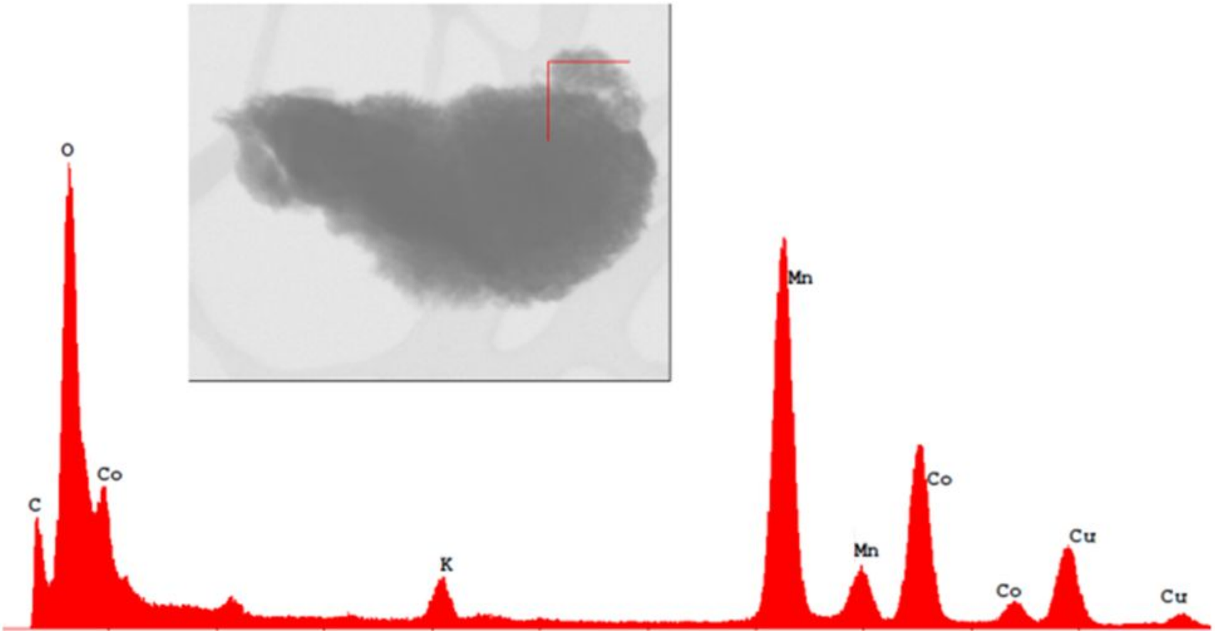

Figure S20. EDS of Co-doped birnessite showing presence of K, Mn, and Co.[10] Copper signals are an artifact of the mounting stage. TEM image shown in inset.

Additional Tables

Table S1. Experimental parameters for GC-TCD and GC-FID

**Method**	**TCD**	**FID**
**Oven**		
Initial Temperature	35 °C	40 °C
Final Temperature	250 °C	250 °C
Temperature Ramp	24 °C/min	10 °C/min
**Front Inlet**		
Mode	Split	Splilt
Temperature	200 °C	200 °C
Split Ratio	4:1	5:1
Split Flow	22.9 mL/min	2.5 mL/min
Total Flow	29.2 mL/min	10.4 mL/min
**Detector**		
Temperature	230 °C	260 °C
Mode	Constant makeup	Constant makeup
H2 Flow		55 mL/min
Air Flow		400 mL/min
Makeup Flow (N2)		25 mL/min
Makeup Flow (He)	2 mL/min	
**Instrument**	**Collumn**	**Column Dimensions**
Agilent 7820A (TCD)	Carbonex 1010 Plot fused silica	30 m x 0.53 mm
Agilent 6890 (FID)	Rt-Q bond Plot fused silica	30 m x 0.53 mm

Table S2. The phase abundance results (wt%) from the Rietveld refinement for the activated all-cobalt catalyst sample. The data are plotted in Figure S10. Unfilled boxes mean the phases were not observed at those temperatures

	Co(hcp)	Co(ccp)	Co_2C	Li_2CO_3	LiOH	Li_2O	C
25 °C	43.7(8)	12.2(4)		11.4(9)	29(1)	4.1(5)	
200	52.4(8)	14.3(4)		13(1)	16(1)	3.9(6)	
250	52.1(8)	14.4(4)		15(1)	15(1)	3.6(6)	
300	43.2(8)	10.8(4)	11.9(4)	18(1)	12.3(5)	3.6(5)	
350	36.1(8)	7.9(4)	17.7(6)	36(1)	2.2(3)		
400	37.7(8)	10.4(4)	3.4(3)	38(1)			11(1)
450	32.3(8)	11.0(4)		32(2)			25(2)
500	31.7(8)	11.1(4)		32(2)			25(2)

Table S3. The phase abundance results (wt%) from the Rietveld refinement for the activated Co-doped birnessite sample. The data are plotted in Figure S10. Unfilled boxes mean the phases were not observed at those temperatures.

	Co(hcp)	Co(ccp)	Co_2C	K_2CO_3	KOH	MnO	C
25 °C	13.3(3)	6.1(2)			10.7(3)	69.9(3)	
200	14.3(3)	4.9(2)		7.2(2)		73.6(3)	
250	14.2(3)	5.0(2)		7.4(2)		73.4(3)	
300	12.9(3)	4.7(2)	0.8(1)	11.8(3)		69.7(3)	
350	9.9(3)	3.6(2)	4.8(1)	12.0(3)		69.7(3)	
400	9.3(3)	3.1(1)	6.1(1)	11.3(2)		70.2(3)	
450	10.2(3)	4.6(2)		7.2(1)		53.3(5)	24.7(7)
500	6.1(2)	2.9(1)		2.9(1)		30.5(3)	57.5(4)

Table S4. Crystallite size estimates for the two metallic Co phases from Rietveld Refinement

	All-cobalt catalyst (nm)		Co-doped MnO catalyst (nm)	
	Co(hcp)	Co(ccp)	Co(hcp)	Co(ccp)
25 °C	22.8(6)	23(1)	7.5(2)	30(3)
200	22.5(6)	21(1)	7.8(3)	43(6)
250	22.4(6)	19(1)	7.8(2)	42(6)
300	24.7(6)	20(1)	7.8(3)	33(4)
350	24.9(6)	23(2)	7.9(3)	29(4)
400	18.6(4)	19(1)	7.9(3)	39(7)
450	16.0(4)	18(1)	5.6(2)	25(2)
500	16.2(4)	20(1)	6.0(2)	19(1)

Table S5. Refinement R_{wp} values in percentage

	All-cobalt system	Co-doped MnO system
25 °C	12.6	7.5
200	12.5	7.9
250	12.3	7.6
300	9.3	7.0
350	8.3	6.1
400	10.7	5.9
450	11.5	6.7
500	11.5	6.0
